# Novel Cinnamaldehyde Hydrazones: Design, In Silico Evaluation, Synthesis, and Cytotoxic Activity

**DOI:** 10.3390/molecules31101701

**Published:** 2026-05-17

**Authors:** Boryana Nikolova-Mladenova, Rositsa Mihaylova, Mariyana Atanasova

**Affiliations:** 1 Department of Chemistry, Faculty of Pharmacy, Medical University of Sofia, 2 Dunav Str., 1000 Sofia, Bulgaria; 2 Department of Pharmacology, Pharmacotherapy and Toxicology, Faculty of Pharmacy, Medical University of Sofia, 2 Dunav Str., 1000 Sofia, Bulgaria; rmihaylova@pharmfac.mu-sofia.bg; 3 Centre of Excellence in Informatics and Information and Communication Technologies, Bulgarian Academy of Sciences, Akad. Georgi Bonchev Str., Block 2 and 25A, 1113 Sofia, Bulgaria

**Keywords:** cinnamaldehyde, hydrazones, anticancer activity, leukemia cells, selectivity

## Abstract

(1) Background: Cinnamaldehyde exhibits a broad spectrum of biological activities, and its α,β-unsaturated aldehyde scaffold serves as a versatile platform for the design of hydrazone derivatives with improved pharmacological properties. (2) Methods: In this study, eight cinnamaldehyde-based hydrazones were synthesized via a one-step condensation reaction between cinnamaldehyde and para-substituted acylhydrazides. Prior to synthesis, an in silico assessment of physicochemical, pharmacokinetic, ADME (absorption, distribution, metabolism, elimination), lead-likeness, and drug-likeness properties was conducted using SwissADME, ACD/Labs v. 9.10, and MDL QSAR v2.2.0.0.446 software. Structural characterization by IR, ^1^H NMR, ^13^C NMR, and HR ESI–MS confirmed successful formation of the hydrazone linkage. Cytotoxic activity was evaluated using the MTT assay against selected cancer cell lines. (3) Results: All compounds exhibited favorable lead-like characteristics, including suitable molecular weight, moderate lipophilicity, and acceptable predicted ADME profiles. Biological evaluation revealed moderate, structure-dependent antiproliferative activity with clear cell line selectivity. Among the series, compound CA8 showed the most promising profile, displaying the highest cytotoxic activity against T-cell leukemia KE-37 cells (IC_50_ = 20.3 ± 2.8 μM), comparable to reference drug melphalan (IC_50_ = 21.40 ± 3.9 μM), and the highest selectivity index (≥19.7). Structure–activity analysis suggests that an amino substituent enhances both potency and selectivity. (4) Conclusions: Overall, these findings identify cinnamaldehyde hydrazones as a promising scaffold for anticancer drug development and provide a strong basis for further structural optimization.

## 1. Introduction

Cinnamaldehyde is a naturally occurring aromatic aldehyde extracted from the bark, leaves, and twigs of *Cinnamomum verum*, *C. cassia*, and related species. As the principal component of cinnamon essential oil, it is responsible for the characteristic flavor and aroma of cinnamon. According to U.S. Food and Drug Administration (FDA) cinnamaldehyde is currently classified as safe and well-tolerated in humans (available at https://hfpappexternal.fda.gov/scripts/fdcc/index.cfm?set=FoodSubstances&id=CINNAMALDEHYDE, accessed on 8 April 2026) which underpins its widespread use as a food-additive and in cosmetic, and pharmaceutical formulations. Beyond its organoleptic properties, cinnamaldehyde has emerged as a privileged scaffold in medicinal chemistry. It exhibits a broad-spectrum of pharmacological properties, including antioxidant activity [1,2,3], neuroprotective properties [4,5,6], anti-inflammatory [7,8], antimicrobial [9,10], antifungal [11], antibacterial [12], antidiabetic [13,14,15], and anticancer [16,17,18,19] activity.

Cinnamaldehyde displays significant antioxidant potential through both direct radical scavenging and indirect modulation of antioxidant signaling pathways [1]. Recently studies demonstrated that cinnamaldehyde derivatives exhibit strong antioxidant activity across multiple assays, including DPPH, superoxide, hydroxyl radical, and nitric oxide scavenging tests [2,3]. Results revealed that cinnamaldehyde has the same free radical-scavenging properties as standard vitamin C. Moreover, cinnamaldehyde displays neuroprotective effects in Alzheimer’s disease and Parkinson’s disease animal models by modulating neuroinflammation, suppressing oxidative stress, and improving the synaptic connection [4,5,6]. Cinnamaldehyde modulates inflammatory mediators such as nuclear factor kappa B (NF-κB) and mitogen-activated protein kinase (MAPK) pathways, which contributes to its anti-inflammatory properties [7,8]. It also possesses strong antimicrobial activity against a wide range of pathogenic microorganisms [9,10]. Cinnamaldehyde demonstrates strong antibacterial and antifungal properties against a variety of pathogens, making it useful in both medical and food preservation applications [11,12]. Several investigations indicate Cinnamaldehyde’s anti-hyperglycemic, antidiabetic, and metabolic effects [13,14,15]. It significantly modulates glucose metabolism and improves glucose levels in blood by increasing insulin sensitivity and by inhibiting *α*-amylase and *α*-glucosidase enzymes activity [13,14]. Cinnamaldehyde exhibits promising anticancer properties by modulating various cellular processes involved in tumor growth and progression [16,17]. It has an antiproliferative effect against cancers that originate in various parts of the body, like blood, brain, breast, bladder, cervical, colon, lung, liver, prostate, renal, and skin cancer [16,17]. Its anticancer effects are mediated through several molecular mechanisms, including inhibition of cell proliferation, induction of apoptosis and cell cycle arrest [16,17,18]. Cinnamaldehyde shows relatively low toxicity toward normal cells while targeting cancer cells. This selectivity highlights cinnamaldehyde’s potential as therapeutic agent and makes it a promising candidate for developing safer anticancer therapies [19].

**Figure 1:**
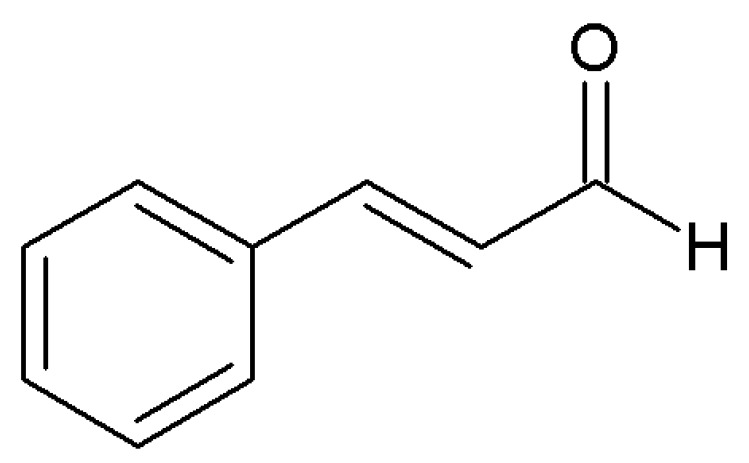
Structure of cinnamaldehyde.

Structurally, cinnamaldehyde (3-phenyl-2-propenal) is a naturally occurring α,β-unsaturated aromatic aldehyde that serves as an attractive scaffold for the development of new bioactive molecules (Figure 1). Its conjugated phenyl-propenal system displays pronounced electrophilic character, which largely determines its chemical reactivity and biological profile, including its frequently reported cytotoxic effects.

Condensation of cinnamaldehyde with various molecules is commonly employed to expand structural diversity and generate new compounds with enhanced drug-like characteristics and targeted biological activity. Through this approach, several types of derivatives have been synthesized, including Schiff bases, hydrazones, and chalcones [20,21,22,23,24,25,26,27,28]. Among the resulting classes, hydrazones are the most extensively studied because of their structural flexibility, tunable electronic properties, and capacity to interact with a wide range of biological targets. Recently, antiparasitic activities of cinnamaldehyde acylhydrazones were evaluated against two clinically important protozoan parasites [20]. Some of the compounds showed strong and selective antitoxoplasmal activity against *T. gondii* parasites achieving low micromolar IC_50_ values and favorable selectivity indices. Another study reports design and preparation of cinnamaldehyde-based hydrazones by condensation reactions between cinnamaldehyde and appropriate phenylhydrazine hydrochlorides [21]. All compounds exhibited measurable antifungal activity at 50 μg/mL, and some showed >50% inhibition, demonstrating broad-spectrum potential. Similar hydrazones have been used as bidentate ligands and form octahedral complexes with transition-metal ions such as Cu(II), Ni(II), Mn(II), Co(II), and Fe(II) [22]. Complexes, together with the free hydrazone ligands, display measurable antimicrobial activity against *Bacillus subtilis* and *Escherichia coli*, with all compounds tested being more active against the Gram-positive bacteria *Bacillus subtilis* than against the Gram-negative bacteria *E. coli* [22]. Furthermore, the effect of cinnamaldehyde-based hydrazones on digestive enzymes lipase and α-amylase also has been investigated [23]. Compounds demonstrate a clear and meaningful inhibitory effect on both digestive enzymes, with several compounds outperforming the reference drugs [23]. Another study unveiled the significance of the cinnamaldehyde hydrazones as cathepsin B inhibitors at nanomolar concentrations [24]. Santos et al. [25] demonstrated that some cinnamaldehyde hydrazones exhibited promising in silico profile and high cytotoxic activity against leukemic (HL-60) and glioblastomas (SF-295) cell lines. The results indicate that specific structural features, particularly substitution in aromatic nuclei and extended conjugation, enhance biological response, and may serve as useful starting points for further optimization [25]. A recent study [26] reported that pyrrolizine-based cinnamaldehyde Schiff bases exhibit strong cytotoxic activity together with favorable ADME (absorption, distribution, metabolism, elimination) characteristics. The acylhydrazide moiety further contributes to improved molecular stability, enhanced lipophilicity, and greater structural diversity, all of which are key parameters in optimizing biological performance. For these reasons, integrating the cinnamaldehyde core with the N-acylhydrazone is anticipated to produce derivatives with enhanced biological activity.

A variety of cinnamaldehyde-derived hydrazones have been reported, but the studies are highly heterogeneous in terms of structural design, substitution patterns, and biological evaluation protocols. Such variability limits direct comparison across studies and complicates the establishment of consistent structure–activity relationships. Consequently, despite the recognized biological potential of cinnamaldehyde hydrazones, the currently available data do not support the identification of a well-defined lead compound suitable for systematic optimization. In this context, a research approach aimed at exploring the chemical space around this scaffold is warranted to identify emerging structure–activity trends and potential lead candidates.

Guided by these considerations, we designed a series of eight hydrazones obtained by coupling cinnamaldehyde with a set of para-substituted benzhydrazides. This substitution pattern was chosen to combine the well-documented biological potential of cinnamaldehyde with the versatile pharmacophoric features of hydrazone bonds. Substituents at the para position can significantly modulate the electron distribution, lipophilicity, and steric properties of the molecule without disrupting the underlying hydrazone backbone. Prior to synthesis, all proposed structures were evaluated in silico for drug likeness, critical physicochemical properties, ADME characteristics, and pharmacokinetic parameters. Computational screening indicated favorable properties, therefore the compounds were synthesized and subsequently assessed for their cytotoxic activity.

## 2. Results

### 2.1. Design of Novel Cinnamaldehyde Hydrazones and In Silico Evaluation

A series of eight cinnamaldehyde hydrazones was designed by combination of cinnamaldehyde with various acylhydrazides with different substituents on 4th position in hydrazide nuclei. The selected hydrazides incorporate molecular fragments found in numerous biologically active compounds, which are essential for their functional activity (Table 1). The unsubstituted derivative, cinnamaldehyde benzoylhydrazone (CA1), bearing a hydrogen atom at the para position of the hydrazide ring, is a previously described compound [25,27] and was therefore adopted as a reference structure and base molecule for this study, against which the impact of structural modifications could be assessed.

Before synthesizing the designed compounds, a comprehensive in silico evaluation of the molecular properties of cinnamaldehyde hydrazones was conducted based on various criteria. This assessment focused on drug likeness, critical physicochemical properties, and ADME (absorption, distribution, metabolism, and excretion) characteristics, including molecular weight, predicted pKa, fraction of ionized species, logP, polar surface area, number of free rotatable bonds, hydrogen-bond donors, hydrogen-bond acceptors, and any violations of Lipinski’s rule of five, aqueous solubility, gastrointestinal absorption, oral bioavailability, bioavailability score, blood–brain barrier (BBB) permeability, potential inhibition of cytochrome P450 enzymes, likelihood of being a P-glycoprotein substrate, drug-likeness, lead-likeness, and synthetic accessibility (Table 2). The evaluations utilized the SwissADME web tool (accessible at https://www.swissadme.ch/ assessed on 14 April 2026) and the ACD/Labs Suite v. 9.10 provided by Advanced Chemistry Development, Inc., Toronto, ON, Canada.

Furthermore, pharmacokinetic parameters were predicted through previously established quantitative structure–pharmacokinetics relationship (QSPkR) models [29,30,31]. They include the fraction of unbound drug in plasma, total clearance, steady-state volume of distribution (Table 2).

The molecular weights (Mw) of the investigated compounds, ranging from 250.30 to 295.29 g/mol, indicate that the cinnamaldehyde hydrazones are more appropriately categorized as lead-like rather than drug-like molecules [32]. This relatively low molecular mass provides sufficient scope for further structural elaboration and optimization aimed at enhancing anticancer potency and selectivity. All compounds exhibit very weak acidic character, with predicted pKa values in the range of 8.47–13.49. Consequently, at physiological pH (7.4), they are expected to exist predominantly in their non-ionized form, as reflected by a negligible fraction of ionized species (f_A_ ≈ 0).

The consensus LogP values for the cinnamaldehyde hydrazones range between 2.33 and 3.58, indicating a moderate lipophilicity. This range is generally considered favorable for drug-like molecules, as it reflects an appropriate balance between aqueous solubility and membrane permeability, supporting both sufficient dissolution and effective passive diffusion across biological membranes. The unsubstituted hydrazone CA1 (LogP = 3.13) serves as a reference point for assessing the influence of substituents at the para position of the hydrazide moiety. Introduction of a methyl group (CA2) leads to an increase in lipophilicity (LogP = 3.33), consistent with its electron-donating and hydrophobic nature. An even more pronounced effect is observed for the chloro-substituted derivative CA3, which exhibits the highest LogP value (3.58), reflecting the combined hydrophobic and electron-withdrawing character of the chlorine atom. In contrast, incorporation of a nitrogen atom in the aromatic ring (CA4) results in a substantial decrease in lipophilicity (LogP = 2.33), likely due to increased polarity and hydrogen bond acceptor capacity. A similar trend is observed for derivatives bearing polar substituents. The hydroxy (CA5, LogP = 2.64) and amino (CA8, LogP = 2.73) groups reduce lipophilicity due to their ability to participate in hydrogen bonding. The nitro-substituted compound CA7 (LogP = 2.44) also shows decreased lipophilicity, which can be attributed to the strong electron-withdrawing and polar nature of the –NO_2_ group. Interestingly, the methoxy derivative CA6 (LogP = 3.05) retains relatively high lipophilicity compared to other electron-donating substituents, which may be explained by the balance between its weak polarity and hydrophobic methyl fragment.

The calculated polar surface area (PSA), ranging from 41.46 to 87.28 Å^2^, with the highest value observed for CA7, bearing the most polar nitro substituent. This is followed by compounds containing progressively less polar groups, namely amino (CA8), hydroxy (CA5), heterocyclic nitrogen (CA4), and methoxy (CA6). This range falls well within the limits generally associated with favorable oral bioavailability (PSA ≤ 140 Å^2^), suggesting good membrane permeability and high intestinal absorption (>90%) [33,34,35,36]. Notably, the three most polar derivatives, CA8, CA7, and CA5, exhibit PSA values within the range typically correlated with central nervous system (CNS) activity (PSA < 60–90 Å^2^), indicating a potential for blood–brain barrier (BBB) penetration [37,38].

Although the molecules possess five or six freely rotatable bonds (six in CA6 and CA7), their overall conformational flexibility is moderated by extended π-conjugation between the aromatic systems and the hydrazone linkage, which contributes to a relatively rigid molecular framework. Furthermore, the presence of one or two hydrogen bond donors (for CA5 and CA8) and two to four hydrogen bond acceptors, in combination with suitable molecular weight and lipophilicity, ensures that all compounds comply with Lipinski’s rule of five, supporting their potential as orally bioavailable candidates [32].

With respect to ADME-related properties, the cinnamaldehyde hydrazones are generally predicted to be soluble, with the exception of CA3 and CA7, which exhibit moderate aqueous solubility. All compounds demonstrate high predicted gastrointestinal (GI) absorption, which is further supported by bioavailability score of approximately 0.55, suggesting a reasonable probability of achieving measurable oral exposure (>10% in rats). Such behavior is consistent with the relatively low fraction of sp^3^-hybridized carbons, a descriptor often associated with favorable early-stage oral performance. Most derivatives are also predicted to be permeable across the blood–brain barrier (BBB), with CA7 representing the only exception. In terms of metabolic interactions, six compounds are predicted to inhibit CYP1A2 and CYP2C19, while CA3 and CA7 may additionally inhibit CYP2C9, suggesting a broader interaction profile. In contrast, CA5 and CA8 are not predicted to inhibit cytochrome P450 enzymes. Notably, none of the compounds are predicted to be substrates of P-glycoprotein. Overall, the series conforms well to lead-like criteria, with molecular weights in the range of 250–350 g/mol, XLogP values generally ≤ 3.5, and ≤7 freely rotatable bonds [39]. However, CA2 and CA3 slightly exceed the preferred lipophilicity threshold, placing them at the margin of the typical lead-like space. Finally, synthetic accessibility scores ranging from 2.67 to 2.84 suggest that these compounds are readily amenable to synthesis.

With respect to the predicted pharmacokinetic parameters [29,30,31], all compounds exhibit low steady-state volumes of distribution (VD_ss_; 0.220–0.406 L/kg). This suggests that the molecules are largely confined to the systemic circulation, with limited distribution into tissues. Such behavior may reflect either substantial plasma protein binding or insufficient lipophilicity to promote extensive tissue partitioning. Notably, compound CA5 shows a VD_ss_ value of 0.220 L/kg, which is close to the volume of extracellular fluid, further supporting its restricted distribution profile. This distribution pattern is characteristic of compounds with limited cellular permeability, such as acidic molecules that preferentially bind to plasma proteins, particularly human serum albumin. In line with this, the predicted fraction of unbound drug in plasma is relatively low (f_u_ ≈ 0.1 for most compounds, with the exception of CA4, f_u_ = 0.209), corresponding to approximately 90% plasma protein binding. This observation is consistent with the well-established tendency of acidic compounds to exhibit strong albumin binding [40]. From a clearance perspective, large-scale analyses indicate that the majority of anionic drugs are typically associated with low systemic clearance (CL < 0.24 L/h/kg), while only a small proportion display high clearance values (CL > 0.96 L/h/kg) [41]. In contrast, the predicted total clearance values for the present series range from 18.153 to 46.443 mL/min/kg (1.09–2.79 L/h/kg), placing most compounds within the high-clearance category (CL ≥ 0.96 L/h/kg).

Overall, the in silico evaluation of physicochemical, ADME, lead-likeness, and pharmacokinetic properties indicates that the newly designed cinnamaldehyde hydrazones possess a well-balanced profile, characterized by an appropriate interplay between hydrophilicity and lipophilicity, favorable molecular size, and acceptable conformational flexibility. These features collectively support adequate solubility, membrane permeability, and oral absorption potential. At the same time, the predicted pharmacokinetic liabilities, particularly the tendency toward high clearance and significant plasma protein binding, highlight key aspects that may require further optimization. Taken together, these findings position the current series as promising lead-like candidates, justifying their prioritization for synthesis and experimental validation.

### 2.2. Synthesis and Characterization of Cinnamaldehyde Hydrazones

**Figure 2:**
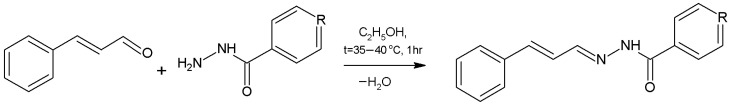
Synthesis of cinnamaldehyde hydrazones.

The cinnamaldehyde hydrazones were prepared via a one-step Schiff base condensation reaction between cinnamaldehyde and a series of eight hydrazides bearing different substituents at the para position, using ethanol as the reaction medium, following previously reported procedures (Figure 2) [42,43,44]. The compounds were obtained as solids in good yields. Hydrazones CA1 and CA4 have been reported previously in the literature [25,27], while the other six hydrazones are newly synthesized.

The obtained hydrazones were isolated in high yields, and their molecular compositions were verified by elemental analysis and high-resolution electrospray ionization mass spectrometry (HR ESI–MS). Structural characterization was carried out using IR, ^1^H NMR, and ^13^C NMR spectroscopy. All corresponding data are provided in the Supplementary Materials.

In the IR spectra, the appearance of a characteristic band in the region 1603–1626 cm^−1^, attributed to C=N stretching vibrations, confirmed the formation of the hydrazone linkage through condensation of the cinnamaldehyde carbonyl group with the acylhydrazides. Absorption bands of medium intensity observed at 3192–3280 cm^−1^ were assigned to NH stretching vibrations. A strong, sharp band at 1626–1673 cm^−1^ corresponded to the carbonyl (C=O) group, indicating that the compounds predominantly exist in the keto form in the solid state.

Further structural insights were obtained from ^1^H and ^13^C NMR spectra recorded in DMSO-d_6_. The azomethine proton (HC=N) appeared as signals in the range of 8.19–8.38 ppm in the ^1^H NMR spectra, while the NH protons were observed as broad singlets between 11.35 and 12.03 ppm. The ^13^C NMR spectra supported these findings, showing signals for the azomethine carbon atoms in the region 138.28–150.57 ppm, while the carbonyl carbon resonances were detected at 152.76–163.87 ppm.

### 2.3. In Vitro Cytotoxic Activity and Selectivity

The anticancer potential of the newly synthesized cynnamaldehyde hydrazone derivatives was evaluated across a diverse panel of human malignant cell lines representing different tissue origins and molecular profiles. The study included several in vitro leukemia models, namely acute promyelocytic leukemia (HL-60), chronic myeloid leukemia (K-562), and T-cell leukemia (KE-37). In addition, two principal subtypes of human breast adenocarcinoma were examined: hormone-responsive MCF-7 cells, characterized by high expression of progesterone (PR) and estrogen receptor α (ERα), and the triple-negative MDA-MB-231 cell line, which lacks ERα, PR, and human epidermal growth factor receptor 2 (HER2).

**Figure 3:**
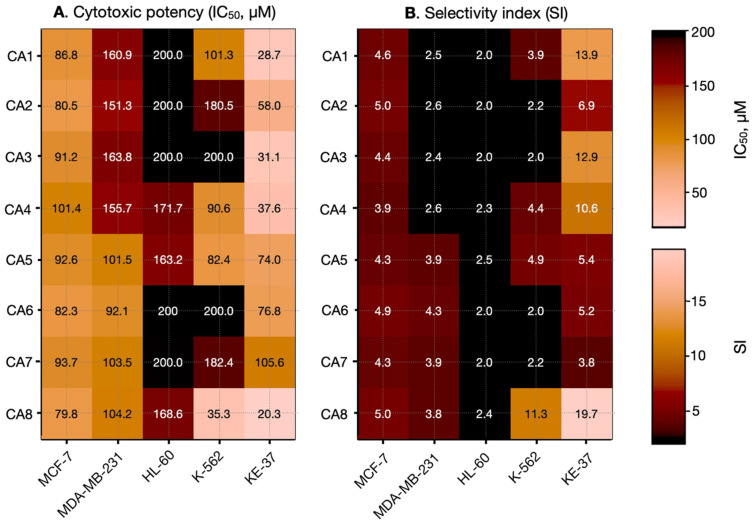
Cytotoxic potency and selectivity profile of newly synthesized cinnamaldehyde hydrazones across cancer cell lines of different origin. (**A**) Heatmap representation of cytotoxic activity expressed as IC_50_ values (µM) against breast cancer cell lines (MCF-7, MDA-MB-231), leukemia cell lines (HL-60, K-562, KE-37), where lighter colors indicate higher potency (lower IC_50_ values) and darker colors correspond to reduced activity. (**B**) Heatmap of selectivity indices (SI), calculated as the ratio of IC_50_ values in non-malignant HEK-293 cells to those in the respective cancer cell lines, with lighter colors indicating higher selectivity.

To assess the selectivity of the compounds toward cancer cells, their effects were also tested on non-tumorigenic human embryonic kidney cells (HEK-293). The IC_50_ values obtained from these experiments, as well as the selectivity indices, are presented in Table 3 and Figure 3.

The cytotoxic evaluation of the newly synthesized cinnamaldehyde hydrazones demonstrates a moderate yet distinctly structure-dependent antiproliferative activity, without revealing a universal structure–activity relationship (SAR) applicable across all tested cell lines. Instead, the observed biological effects are highly cell line-specific, indicating that multiple molecular mechanisms and cellular contexts influence compound efficacy.

Among the tested derivatives, CA8 emerges as the most potent compound, particularly against the leukemic KE-37 cell line, with an IC_50_ of 20.3 ± 2.8 μM—comparable to that of the reference drug melphalan (IC_50_ = 21.40 ± 3.9 μM). It also demonstrates notable activity against K-562 cells, with an IC_50_ of 35.30 ± 4.1 μM, which is in a similar range to that of the reference compound (28.2 ± 7.1 μM). Importantly, CA8 is associated with the highest selectivity index, almost 20, suggesting a favorable balance between cytotoxic potency and selectivity toward malignant cells. This profile highlights its potential as a lead structure for further optimization. In contrast, compounds such as CA6 and CA7 exhibit comparatively weak cytotoxic effects, especially in epithelial models, which is further reflected in their lower selectivity indexes, indicating reduced therapeutic relevance.

Although no global SAR trend can be established, distinct cell line-dependent patterns emerge, particularly in KE-37 cells. Within this model, the introduction of OH, OCH_3_, and NO_2_ substituents (CA5-CA7) results in a 2–3-fold reduction in activity, suggesting that these functional groups may unfavorably affect electrophilicity, intracellular reactivity, or membrane permeability. This reduction in potency is paralleled by a decline in selectivity, indicating that such substitutions may compromise both efficacy and specificity.

Conversely, the presence of an NH_2_ group in the benzoyl fragment enhances cytotoxic activity, most notably in KE-37, but also in K-562 cells. This modification is also associated with improved selectivity, suggesting that the amino group may facilitate stronger or more specific interactions with intracellular targets, potentially via hydrogen bonding or modulation of physicochemical properties such as polarity and solubility. These findings point to a favorable role of amino substitution in optimizing both activity and selectivity.

Regarding cell line sensitivity, KE-37 emerges as the most responsive model, displaying both lower IC_50_ values and higher selectivity indexes across several compounds. In contrast, HL-60 appears relatively resistant, with consistently higher IC_50_ values and poor selectivity, which may reflect intrinsic cellular defense mechanisms, such as enhanced detoxification capacity or altered apoptotic signaling.

In epithelial models, a clear divergence is observed. The triple-negative MDA-MB-231 cell line shows consistently weak responsiveness and low selectivity, consistent with its aggressive and treatment-resistant phenotype. Conversely, the hormone-responsive MCF-7 cell line demonstrates moderate and relatively uniform sensitivity across the compound series, suggesting a more predictable response, albeit with moderate selectivity compared to leukemic models.

## 3. Discussion

Design and synthesis of biologically active small molecules remain a central objective in medicinal chemistry, particularly in the development of novel anticancer agents. Among the various structural classes explored, hydrazone derivatives have attracted considerable attention due to their broad spectrum of pharmacological activities and favorable synthetic accessibility. The present study integrates rational design, in silico profiling, chemical synthesis, and biological evaluation to explore the potential of cinnamaldehyde hydrazones as anticancer agents. The design strategy, based on combining the cinnamaldehyde scaffold with substituted acylhydrazides, successfully generated a structurally coherent series of lead-like molecules with desired physicochemical and biological properties. By varying the nature of the para-substituents—from electron-donating to electron-withdrawing groups—we aim to establish structure–activity relationships.

The in silico analysis indicates that all compounds are lead-like molecules, characterized by a relatively low molecular weight, moderate lipophilicity, and compliance with Lipinski’s criteria. These features are advantageous for early-stage drug discovery, as they provide flexibility for further structural optimization. The results for LogP values demonstrate that the lipophilicity of cinnamaldehyde hydrazones is strongly influenced by the electronic nature and polarity of the substituents at the para position. Hydrophobic and weakly polar groups (e.g., –CH_3_, –Cl) increase LogP values, whereas strongly polar or hydrogen bond-forming substituents (e.g., –OH, –NH_2_, –NO_2_, and heteroaromatic nitrogen) decrease them. These findings suggest that fine-tuning of substituents can be used to optimize ADME properties such as membrane permeability and bioavailability.

The biological evaluation reveals a moderate but clearly structure-dependent cytotoxic activity. Importantly, the absence of a universal structure–activity relationship suggests that the biological effects are governed by complex and possibly multiple mechanisms of action, which may vary depending on the cellular types. Among the tested compounds, CA8 emerges as the most promising candidate, demonstrating both enhanced potency and selectivity, particularly against leukemia cell lines (KE-37 and K-562). The improved performance of CA8 may be attributed to the presence of the amino group, which likely enhances target interactions through hydrogen bonding and modulates physicochemical properties such as polarity and solubility. The observed cell line-specific responses further emphasize the complexity of the biological activity. The heightened sensitivity of KE-37 cells suggests that these compounds may preferentially target pathways relevant to T-cell leukemia, whereas the resistance observed in HL-60 and MDA-MB-231 cells may reflect intrinsic differences in cellular metabolism, drug transport, or apoptotic regulation. The moderate and consistent response in MCF-7 cells indicates a more uniform mechanism of action in hormone-responsive breast cancer, although with limited selectivity.

These findings suggest that both electronic effects and hydrogen bonding capabilities of substituents play a critical role in modulating anticancer activity, while cellular context determines the ultimate biological outcome. Beyond these factors, the hydrazone linkage itself may contribute to activity through participation in biologically relevant interactions. Acylhydrazones are known to exhibit metal-chelating properties, raising the possibility that the present compounds could interact with biologically important metal ions (e.g., Fe^2+^, Cu^2+^, Zn^2+^), thereby perturbing metal homeostasis, promoting reactive oxygen species (ROS) generation, or inhibiting metalloenzymes involved in tumor progression [45,46,47]. Although such interactions were not explicitly evaluated in the current study, they represent a plausible mechanistic hypothesis that warrants further investigation.

In parallel, the chemical stability of the hydrazone linkage is an important consideration when interpreting the observed cytotoxic effects. Aromatic hydrazones generally exhibit enhanced stability relative to their aliphatic counterparts due to conjugation with adjacent aromatic and carbonyl systems, which reduces susceptibility to hydrolytic cleavage. In the present series, the extended conjugation provided by the cinnamaldehyde-derived α,β-unsaturated system is expected to further stabilize the azomethine bond. Systematic stability studies in biologically relevant media (e.g., buffered solutions, serum-containing environments, and plasma) will be essential in future work to determine whether compound integrity or potential hydrolysis products contribute to the observed biological activity.

The results underscore the importance of fine-tuning both molecular structure and target cell specificity, with selectivity emerging as a critical parameter alongside cytotoxic potency in the development of cinnamaldehyde-based anticancer agents.

In silico profiling and in vitro evaluation identify CA8 as the most promising compound in the series. It exhibits a balanced ADME profile, including favorable lipophilicity and solubility, high predicted gastrointestinal absorption, no predicted CYP450 inhibition, and compliance with lead-likeness criteria, together with good synthetic accessibility. These properties are consistent with its strong antiproliferative activity and highest selectivity index, particularly against KE-37 and K-562 leukemia cell lines, while maintaining low toxicity toward non-cancerous cells. Structurally, the amino substituent in CA8 is likely responsible for its enhanced activity, contributing to improved hydrogen bonding with biological targets and an optimal balance of polarity and permeability. Compared with the other derivatives, CA8 therefore represents the best compromise between potency, selectivity, and drug-likeness.

Overall, these results support CA8 as a privileged lead candidate within this series. Its combined biological and pharmacokinetic profile highlights its potential for further development, and it warrants continued SAR-guided optimization aimed at improving metabolic stability and target specificity while preserving its favorable pharmacological properties.

## 4. Materials and Methods

### 4.1. Materials

All reagents used in the study were of analytical grade. Cinnamaldehyde, benzhydrazide, and its substituted derivatives (4-methyl-, 4-hydroxy-, 4-methoxy-, 4-nitro-, and 4-amino-benzhydrazide), as well as isoniazid and 96% ethanol, were obtained from Merck/Sigma-Aldrich (Darmstadt, Germany). Elemental composition (carbon, nitrogen, and hydrogen) was determined using a Euro EA 3000-Single analyzer (EuroVector SpA, Milan, Italy). The melting points were determined using a Buchi B-540 apparatus (Flawil, Switzerland). Infrared (IR) spectra were recorded on a Nicolet iS10 FT-IR spectrometer equipped with a Smart iTR accessory. ^1^H and ^13^C NMR spectra were recorded on a Bruker Avance 600 spectrometer (Rheinstetten, Germany), using deuterated dimethyl sulfoxide (DMSO-d_6_) as solvent and tetramethylsilane (TMS) as an internal reference. Chemical shifts (δ) were expressed in parts per million (ppm) and coupling constants (J) in Hertz (Hz). Signal multiplicities were designated as follows: s (singlet), d (doublet), t (triplet), and m (multiplet).

### 4.2. Synthesis and Characterization

A solution of cinnamaldehyde (0.01 mol) in 96% ethanol (25 mL) was gradually added, under constant stirring, to a solution of the respective benzhydrazide (0.01 mol) prepared in 50% aqueous ethanol (75 mL). The reaction mixtures were stirred and maintained at 40 °C for 1 h. After completion, the solutions were allowed to cool and kept at room temperature for 168 h, during which crystalline products formed. The precipitated hydrazones were isolated by filtration and dried in a vacuum desiccator for 72 h.

#### 4.2.1. N′-((1E,2E)-3-phenylallylidene)benzohydrazide (CA1)

Yield: 86%; pale yellow solid; m.p.: 190.5–191.5 °C; IR (ν cm^−1^): 3263 (NH), 1644 (C=O), 1622 (C=N), 1579 (C-NH). ^1^H NMR (600 MHz, DMSO-d_6_) δ ppm: 11.76 (s, 1H), 8.25 (dd, *J* = 6.5, 1.9 Hz, 1H), 7.90 (m, 2H), 7.64 (m, 2H), 7.60 (t, *J* = 7.3 Hz, 1H), 7.53 (t, *J* = 7.6 Hz, 2H), 7.41 (t, *J* = 7.6 Hz, 2H), 7.34 (t, *J* = 7.3 Hz, 1H), 7.07 (m, 2H). ^13^C NMR (151 MHz, DMSO-d_6_) δ ppm: 163.52, 150.28, 139.56, 136.36, 133.85, 132.22, 129.32, 128.95, 128.07, 127.57, 126.12. HR ESI–MS m/z: 251.1172 [M + H]^+^. Calculated for C_16_H_14_N_2_O: C, 76.78; H, 5.64; N, 11.19. Found: C, 76.89; H, 5.53; N, 11.28.

#### 4.2.2. 4-Methyl-N′-((1E,2E)-3-phenylallylidene)benzohydrazide (CA2)

Yield: 83%; pale yellow solid; m.p.: 230.5–232.5 °C; IR (ν cm^−1^): 3193 (NH), 1626 (C=O), 1609 (C=N), 1570 (C-NH). ^1^H NMR (600 MHz, DMSO-d_6_) δ ppm: 11.68 (s, 1H), 8.24 (d, *J* = 7.8 Hz, 1H), 7.82 (d, *J* = 7.8 Hz, 2H), 7.64 (m, 2H), 7.41 (m, 2H), 7.34 (dd, *J* = 7.7, 5.5 Hz, 3H), 7.06 (m, 2H), 2.38 (s, 3H). ^13^C NMR (151 MHz, DMSO-d_6_) δ ppm: 163.30, 149.97, 142.26, 139.33, 136.44, 131.02, 129.47, 129.32, 128.68, 128.12, 127.57, 126.24, 121.50. HR ESI–MS m/z: 265.1329 [M + H]^+^. Calculated for C_17_H_16_N_2_O: C, 77.25; H, 6.10; N, 10.60. Found: C, 77.43; H, 6.24; N, 10.72.

#### 4.2.3. 4-Chloro-N′-((1E,2E)-3-phenylallylidene)benzohydrazide (CA3)

Yield: 89%; pale yellow solid; m.p.: 219–221 °C; IR (ν cm^−1^): 3267 (NH), 1655 (C=O), 1624 (C=N), 1589 (C-NH). ^1^H NMR (600 MHz, DMSO-d_6_) δ ppm: 11.81 (s, 1H), 8.24 (t, *J* = 4.3 Hz, 1H), 7.93 (m, 2H), 7.63 (m, 4H), 7.41 (m, 2H), 7.35 (m, 1H), 7.08 (d, *J* = 4.7 Hz, 2H). ^13^C NMR (151 MHz, DMSO-d_6_) δ ppm: 162.38, 150.57, 139.78, 137.02, 136.35, 132.59, 130.03, 129.37, 129.31, 129.04, 127.61, 126.05. HR ESI–MS m/z: 285. 0784 [M + H]^+^. Calculated for C_16_H_13_ClN_2_O: C, 67.49; H, 4.60; N, 9.84. Found: C, 67.66; H, 4.52; N, 10.02.

#### 4.2.4. N′-((1E,2E)-3-phenylallylidene)isonicotinohydrazide (CA4)

Yield: 86%; pale yellow solid; m.p.: 200–202 °C; IR (ν cm^−1^): 3230 (NH), 1673 (C=O), 1624 (C=N), 1599 (C-NH). ^1^H NMR (600 MHz, DMSO-d_6_) δ ppm: 11.96 (s, 1H), 8.79 (m, 2H), 8.25 (dd, *J* = 7.3, 1.3 Hz, 1H), 7.82 (m, 2H), 7.65 (m, 2H), 7.41 (m, 2H), 7.35 (m, 1H), 7.11 (m, 2H). ^13^C NMR (151 MHz, DMSO-d_6_) δ ppm: 161.12, 138.87, 136.47, 130.13, 129.29, 129.20, 127.49, 126.34, 124.34, 115.46. HR ESI–MS m/z: 252.1127 [M + H]^+^. Calculated for C_15_H_13_N_3_O: C, 71.70; H, 5.21; N, 16.72. Found: C, 71.85; H, 5.16; N, 16.49.

#### 4.2.5. 4-Hydroxyl-N′-((1E,2E)-3-phenylallylidene)benzohydrazide (CA5)

Yield: 84%; pale yellow solid; m.p.: 231–233 °C; IR (ν cm^−1^): 3280 (NH), 1646 (C=O), 1604 (C=N), 1579 (C-NH). ^1^H NMR (600 MHz, DMSO-d_6_) δ ppm: 11.54 (s, 1H), 10.12 (s, 1H), 8.21 (m, 1H), 7.79 (m, 2H), 7.62 (m, 2H), 7.40 (t, *J* = 7.6 Hz, 2H), 7.33 (m, 1H), 7.06 (m, 2H), 6.86 (m, 2H). ^13^C NMR (151 MHz, DMSO-d_6_) δ ppm: 161.12, 138.87, 136.47, 130.13, 129.29, 129.20, 127.49, 126.34, 124.34, 115.46. HR ESI–MS m/z: 267.1122 [M + H]^+^. Calculated for C_16_H_14_N_2_O_2_: C, 72.17; H, 5.30; N, 10.52. Found: C, 72.35; H, 5.23; N, 10.78.

#### 4.2.6. 4-Methoxy-N′-((1E,2E)-3-phenylallylidene)benzohydrazide (CA6)

Yield: 90%; pale yellow solid; m.p.: 212–214 °C; IR (ν cm^−1^): 3192 (NH), 1632 (C=O), 1603 (C=N), 1575 (C-NH). ^1^H NMR (600 MHz, DMSO-d_6_) δ ppm: 11.63 (s, 1H), 8.24 (d, *J* = 8.1 Hz, 1H), 7.90 (d, *J* = 8.4 Hz, 2H), 7.63 (m, 2H), 7.40 (m, 2H), 7.34 (m, 1H), 7.06 (m, 4H), 3.84 (s, 3H). ^13^C NMR (151 MHz, DMSO-d_6_) δ ppm: 163.87, 149.61, 139.09, 136.44, 130.00, 129.30, 129.24, 128.66, 127.52, 126.26, 125.90, 114.18, 55.90. HR ESI–MS m/z: 281.1278 [M + H]^+^. Calculated for C_17_H_16_N_2_O_2_: C, 72.84; H, 5.75; N, 9.99. Found: C, 73.07; H, 5.59; N, 10.18.

#### 4.2.7. 4-Nitro-N′-((1E,2E)-3-phenylallylidene)benzohydrazide (CA7)

Yield: 85%; pale yellow solid; m.p.: 241–242 °C; IR (ν cm^−1^): 3197 (NH), 1634 (C=O), 1621 (C=N), 1538 (C-NH). ^1^H NMR (600 MHz, DMSO-d_6_) δ ppm: 12.03 (s, 1H), 8.38 (m, 2H), 8.27 (m, 1H), 8.15 (m, 2H), 7.65 (m, 2H), 7.41 (dd, *J* = 8.2, 6.7 Hz, 2H), 7.36 (m, 1H), 7.11 (m, 2H). ^13^C NMR (151 MHz, DMSO-d_6_) δ ppm: 161.83, 151.38, 149.73, 140.35, 139.56, 136.29, 129.64, 129.49, 129.33, 127.69, 125.89, 124.11. HR ESI–MS m/z: 296.1023 [M + H]^+^. Calculated for C_16_H_13_N_3_O_3_: C, 65.08; H, 4.44; N, 14.23. Found: C, 65.29; H, 4.53; N, 14.39.

#### 4.2.8. 4-Amino-N′-((1E,2E)-3-phenylallylidene)benzohydrazide (CA8)

Yield: 82%; pale yellow solid; m.p.: 258–260 °C; IR (ν cm^−1^): 3277 (NH), 1655 (C=O), 1624 (C=N), 1578 (C-NH). ^1^H NMR (600 MHz, DMSO-d_6_) δ ppm: 11.35 (s, 1H), 8.19 (d, *J* = 9.0 Hz, 1H), 7.66 (m, 2H), 7.61 (m, 2H), 7.40 (m, 2H), 7.32 (m, 1H), 7.02 (m, 2H), 6.59 (m, 2H), 5.78 (s, 2H). ^13^C NMR (151 MHz, DMSO-d_6_) δ ppm: 152.76, 138.28, 136.58, 129.30, 129.10, 127.44, 126.53, 119.97, 113.06. HR ESI–MS m/z: 266.1281 [M + H]^+^. Calculated for C_16_H_15_N_3_O: C, 72.43; H, 5.70; N, 15.84. Found: C, 72.68; H, 5.85; N, 15.99.

### 4.3. Cell Lines and Culture Conditions

The biological evaluation was performed on a panel of human cancer cell lines, including three leukemic models: HL-60 (acute myeloid leukemia), K-562 (chronic myeloid leukemia), and KE-37 (T-cell leukemia, two breast cancer cell lines: MDA-MB-231 (estrogen receptor-negative) and MCF-7 (estrogen receptor-positive), as well as normal human embryonic kidney cells (HEK-293). All cell lines were obtained from the German Collection of Microorganisms and Cell Cultures (DSMZ, Braunschweig, Germany).

Adherent tumor cell lines (MDA-MB-231 and MCF-7) were cultured as monolayers in RPMI-1640 medium supplemented with 10% fetal bovine serum (FBS), non-essential amino acids, 1 mM sodium pyruvate, and 10 μg/mL human insulin. The remaining cell lines were grown in suspension under standard conditions in RPMI-1640 medium containing 10% FBS and 2 mM L-glutamine. All cultures were maintained at 37 °C in a humidified atmosphere with 5% CO_2_. To preserve logarithmic growth, cells were regularly subcultured two to three times weekly by dilution with fresh medium.

### 4.4. Mosmann’s MTT Test for Cytotoxicity Assessment

The cytotoxic effects of the tested compounds were assessed using the MTT assay, based on the reduction in the yellow tetrazolium salt MTT to purple formazan crystals by mitochondrial succinate dehydrogenase in viable cells [48]. Briefly, exponentially growing cells were seeded in 96-well plates at a density of 1 × 10^5^ cells/mL (100 μL per well). After 24 h incubation, cells were treated with different concentrations of the test compounds for 72 h, with at least eight replicates per concentration. Subsequently, 10 μL of MTT solution (10 mg/mL in PBS) was added to each well, followed by 4 h incubation at 37 °C. The formed formazan crystals were dissolved using 100 μL of 5% formic acid in 2-propanol, and absorbance was measured at 580 nm using a microplate reader (Labexim LMR-1).

### 4.5. Data Processing and Statistics

Cell viability was expressed as a percentage relative to untreated controls (considered as 100%). Statistical analysis was performed using Student’s *t*-test, with *p* ≤ 0.05 regarded as statistically significant. IC_50_ values were calculated by non-linear regression analysis of dose–response curves and represent the concentration required to reduce cell viability by 50%. All results are presented as mean ± standard deviation (SD) from at least eight independent experiments.

### 4.6. In Silico Predictions of Pharmacokinetic Parameters

The studied compounds were modeled using BIOVIA Discovery Studio Visualizer (v21.1.0.20298; Dassault Systèmes, San Diego, CA, USA, 2021). Molecular descriptors for the QSPR models [27,28,29] were calculated using MDL QSAR (v2.2.0.0.446; MDL Information Systems, Inc. 2004. San Leandro, CA, USA: MDL Information Systems, Inc.).

## 5. Conclusions

In this study, a series of eight cinnamaldehyde-based hydrazones was designed, synthesized, and biologically evaluated as potential anticancer agents. The integration of in silico predictions with experimental validation demonstrated that these compounds possess favorable lead-like characteristics, including appropriate physicochemical properties and good predicted oral absorption. Biological evaluation revealed moderate, structure-dependent cytotoxic activity with pronounced selectivity toward certain leukemia cell lines. Compound CA8 emerged as the most promising lead, showing improved potency and favorable selectivity, supported by a balanced ADME profile, good synthetic accessibility, and compliance with lead-likeness criteria. It exhibited strong antiproliferative activity and the highest selectivity index against KE-37 and K-562 cell lines, while maintaining low toxicity toward non-cancerous cells. The amino substituent likely enhances activity through improved hydrogen bonding and target interactions while preserving optimal physicochemical properties. Overall, CA8 represents the best balance of potency, selectivity, and drug-likeness, warranting further SAR optimization. Despite the absence of a universal structure–activity relationship, the study identifies key structural features influencing biological performance and underscores the importance of substituent effects.

## Figures and Tables

**Table 1:** Structures of the newly designed cinnamaldehyde hydrazones.

**Structure**	**ID**	**R**
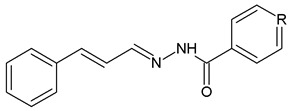	CA1	C–H
	CA2	C–CH_3_
	CA3	C–Cl
	CA4	N
	CA5	C–OH
	CA6	C–OCH_3_
	CA7	C–NO_2_
	CA8	C–NH_2_

**Table 2:** Estimated physicochemical and ADME properties and pharmacokinetic parameters of the cinnamaldehyde-derivative hydrazones.

**Property**	**CA1**	**CA2**	**CA3**	**CA4**	**CA5**	**CA6**	**CA7**	**CA8**
Mw g/mol	250.30	264.32	284.74	251.28	266.29	280.32	295.29	265.31
pK_a_	12.30	12.55	11.93	10.96	8.47	12.43	10.94	13.15
f_A_	0	0	0	0	0	0	0	0
logP	3.13	3.33	3.58	2.33	2.64	3.05	2.73	2.44
PSA Å^2^	41.46	41.46	41.46	54.35	61.69	50.69	87.28	67.48
FRB	5	5	5	5	5	6	6	5
HBD	1	1	1	1	2	1	1	2
HBA	2	2	2	3	3	3	4	2
R5	0	0	0	0	0	0	0	0
Water solubility	Soluble	Soluble	Moderately soluble	Soluble	Soluble	Soluble	Moderately soluble	Soluble
GI absorption	High	High	High	High	High	High	High	High
Oral BA	INSATU	INSATU	INSATU	INSATU	INSATU	INSATU	INSATU	INSATU
BA score	0.55	0.55	0.55	0.55	0.55	0.55	0.55	0.55
BBB permeability	Yes	Yes	Yes	Yes	Yes	Yes	No	Yes
CYP inhibition	1A2, 2C19	1A2, 2C19	1A2, 2C19, 2C9	1A2, 2C19	No	1A2, 2C19	1A2, 2C19, 2C9	No
P-gp substrate	No	No	No	No	No	No	No	No
Drug likeness	Yes	Yes	Yes	Yes	Yes	Yes	Yes	Yes
Lead likeness	Yes	Yes *	Yes *	Yes	Yes	Yes	Yes	Yes
Synthetic Accessibility	2.75	2.84	2.72	2.67	2.67	2.72	2.84	2.70
f_u_	0.146	0.097	0.102	0.209	0.151	0.106	0.136	0.118
CL mL/minh/kg	21.014	20.864	24.010	19.609	46.443	22.190	27.123	18.153
VD_ss_ L/kg	0.403	0.406	0.398	0.396	0.220	0.403	0.377	0.401

Mw—molecular weight, pKa value, f_A_—fraction of the ionized molecules, logP—partition coefficient, PSA—polar surface area, FRB—count of free rotatable bonds, HBDs—hydrogen bond donors, HBAs—hydrogen bond acceptors, R5—count of the violations from Lipinski’s rule of 5, water solubility, GI absorption—gastrointestinal absorption, oral BA—oral bioavailability, INSATU—unsaturation, BA score—bioavailability score, BBB permeability—blood–brain barrier permeability, CYP inhibition—inhibition of CYP enzymes, P-gp substrate—substrate of P-gp, f_u_—fraction of the unbound to plasma protein molecules, CL—total clearance, VD_ss_—steady-state volume of distribution. * XlogP > 3.5.

**Table 3:** Cytotoxicity of the tested compounds after a 72 h exposure (IC_50_, [µM ± SD]) against a panel of human tumor cell lines of different origin.

Cell Line/ Compound	MCF-7	SI _MCF-7_	MDA-MB-231	SI _MDA-MB-231_	HL-60	K-562	KE-37	SI _KE-37_	HEK-293
CA1	86.8 ± 6.2	4.6	160.9 ± 12.5	2.5	>200	101.3 ± 11.2	28.7 ± 1.9	13.9	>400
CA2	80.5 ± 7.4	4.9	151.3 ± 11.4	2.6	>200	180.5 ± 14.8	58.0 ± 7.3	6.9	>400
CA3	91.2 ± 6.9	4.4	163.8 ± 13.2	2.4	>200	>200	31.1 ± 5.2	12.8	>400
CA4	101.4 ± 8.2	3.9	155.7 ± 12.6	2.5	171.7 ± 13.5	90.6 ± 8.9	37.6 ± 3.1	10.6	>400
CA5	92.6 ± 8.6	4.3	101.5 ±9.1	3.9	163.2 ± 14.7	82.4 ± 7.3	74.0 ± 2.1	5.4	>400
CA6	82.3 ± 7.7	4.8	92.1 ± 7.3	4.3	>200	>200	76.8 ± 7.2	5.2	>400
CA7	93.7 ± 6.6	4.2	103.5 ± 10.4	3.8	>200	182.4 ± 12.4	105.6 ± 9.8	3.8	>400
CA8	79.8 ± 5.3	5.0	104.2 ± 11.5	3.8	168.6 ± 10.8	35.3 ± 4.1	20.3 ± 2.8	19.7	>400
melphalan	33.7 ± 4.2	0.7	42.2 ± 3.7	0.6	18.5 ± 2.1	28.2 ± 7.1	21.4 ± 3.9	1.1	24.8 ± 2.9

## Data Availability

Data are contained within the article (and Supplementary Materials).

## Supplementary Materials

The following supporting information can be downloaded at: https://www.mdpi.com/article/10.3390/molecules31101701/s1. Figures S1–S32: HR ESI–MS, IR, ^1^H and ^13^C NMR spectra of the cinnamaldehyde hydrazones.
