# Supplementary material for: Novel Cinnamaldehyde Hydrazones: Design, In Silico Evaluation, Synthesis, and Cytotoxic Activity
Source: Molecules. 2026 May 17;31(10):1701. doi: 10.3390/molecules31101701 (PMC13209825; doi:10.3390/molecules31101701)
Supplement: Supplementary file 1 [file molecules-31-01701-s001.zip › molecules-4293835-supplementary.pdf]

# Supplementary Material

# Novel Cinnamaldehyde Hydrazones: Design, In Silico Evaluation, Synthesis, and Cytotoxic Activity

**Boryana Nikolova-Mladenova [1,*], Rositsa Mihaylova [2], Mariyana Atanasova [1,3,*]**

[1] Department of Chemistry, Faculty of Pharmacy, Medical University of Sofia, 2 Dunav Str., 1000 Sofia, Bulgaria
[2] Department of Pharmacology, Pharmacotherapy and Toxicology, Faculty of Pharmacy, Medical University of Sofia, 2 Dunav Str., 1000 Sofia, Bulgaria; rmihaylova@pharmfac.mu-sofia.bg
[3] Centre of Excellence in Informatics and Information and Communication Technologies, 1113 Sofia, Bulgaria
[*] Correspondence: boriananik@pharmfac.mu-sofia.bg; matanasova@pharmfac.mu-sofia.bg

**Compound CA1**

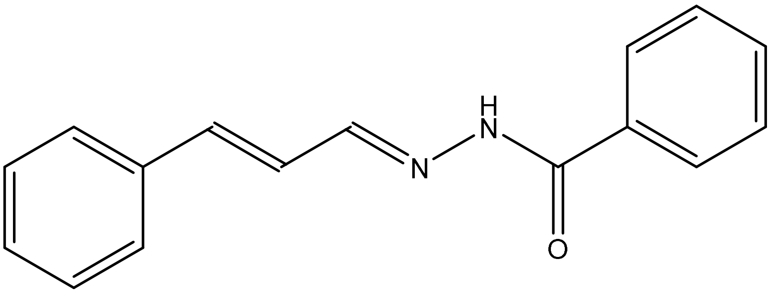


*N'*-((1*E*,2*E*)-3-phenylallylidene)benzohydrazide

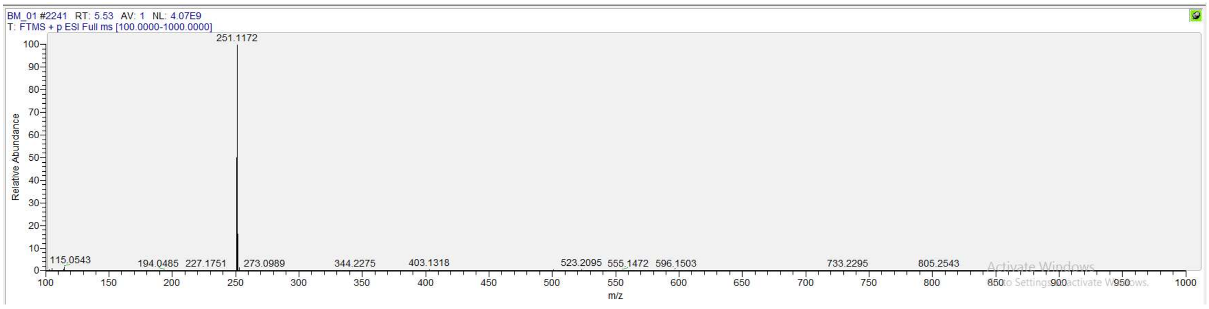


Figure S1. HR ESI–MS spectra of the compound **CA1**.

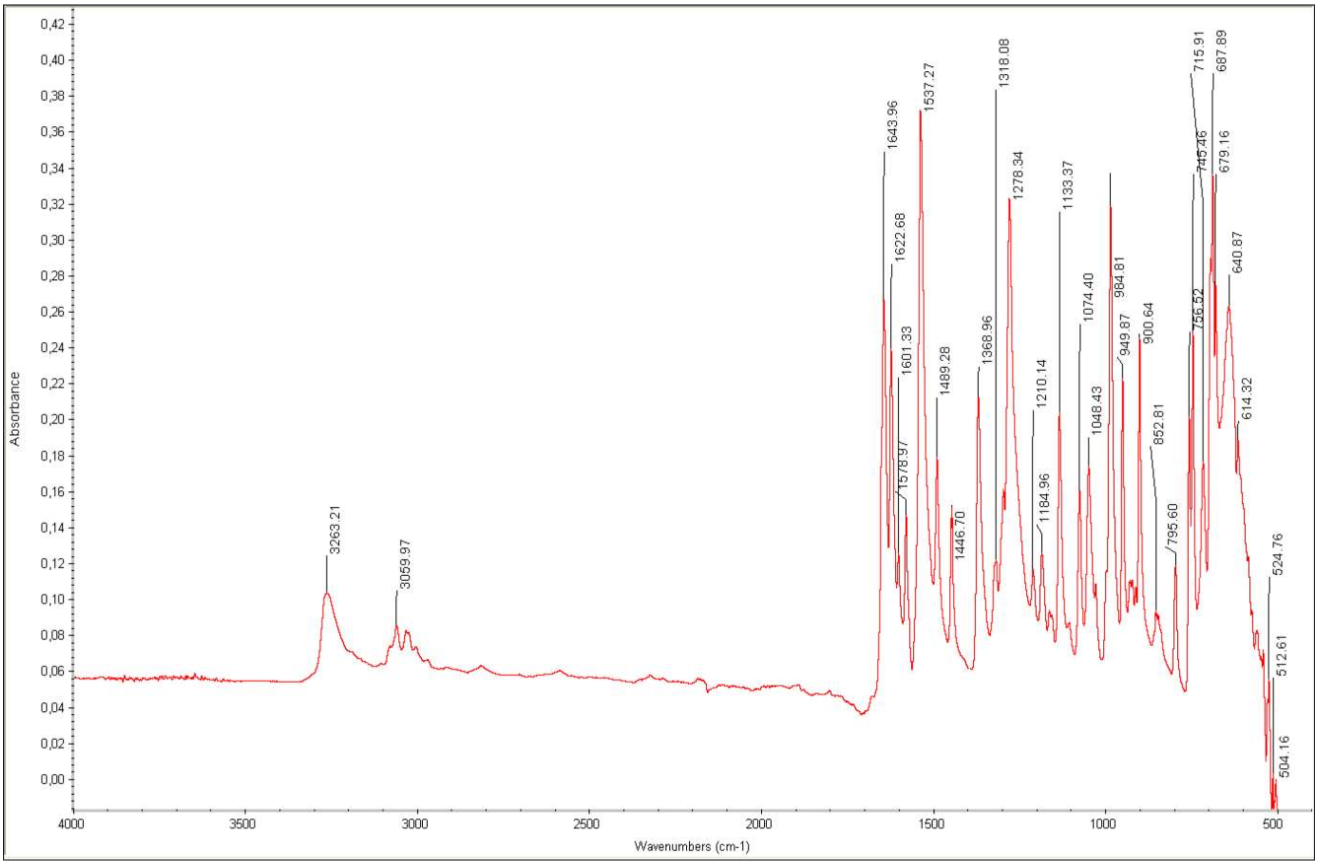


Figure S2. IR spectra of the compound **CA1**.

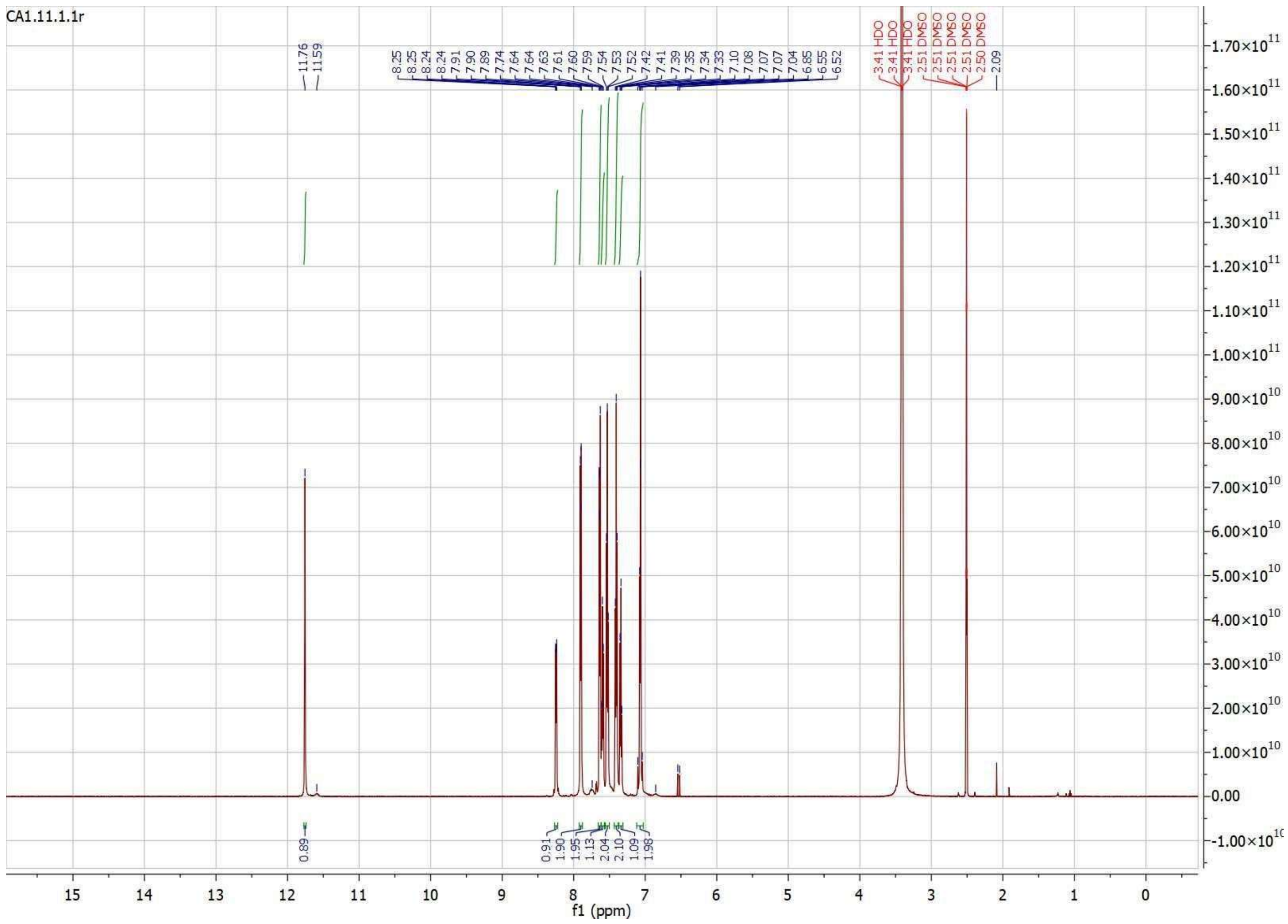

Figure S3. $^{1}$H NMR spectra of the compound **CA1**.

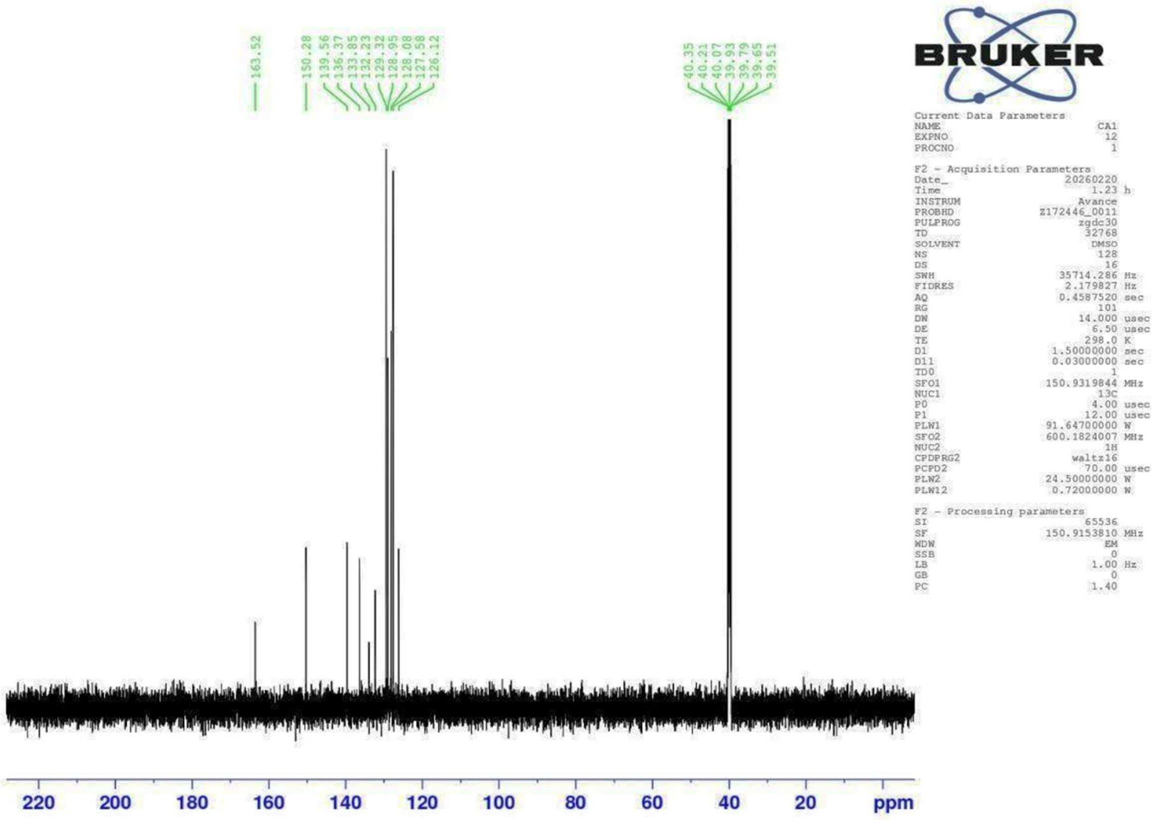


Figure S4. $^{13}$C NMR spectra of the compound **CA1**.

**Compound CA2**

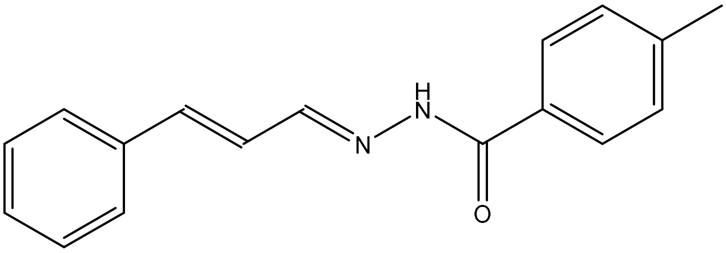


4-methyl-*N'*-((1*E*,2*E*)-3-phenylallylidene)benzohydrazide

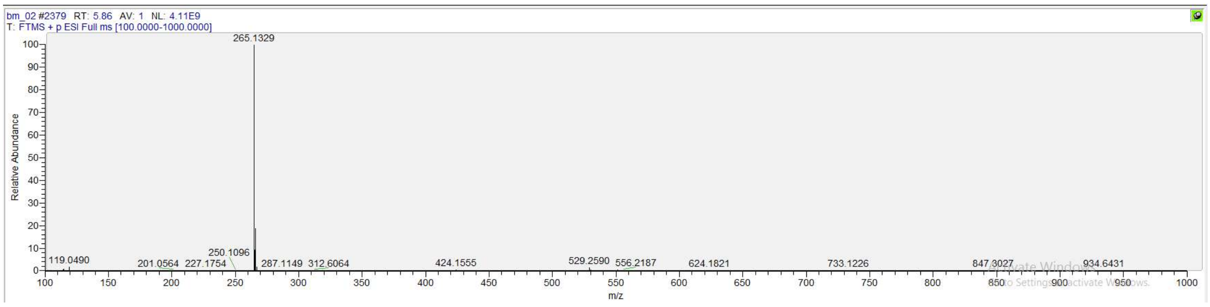


Figure S5. HR ESI–MS spectra of the compound **CA2**.

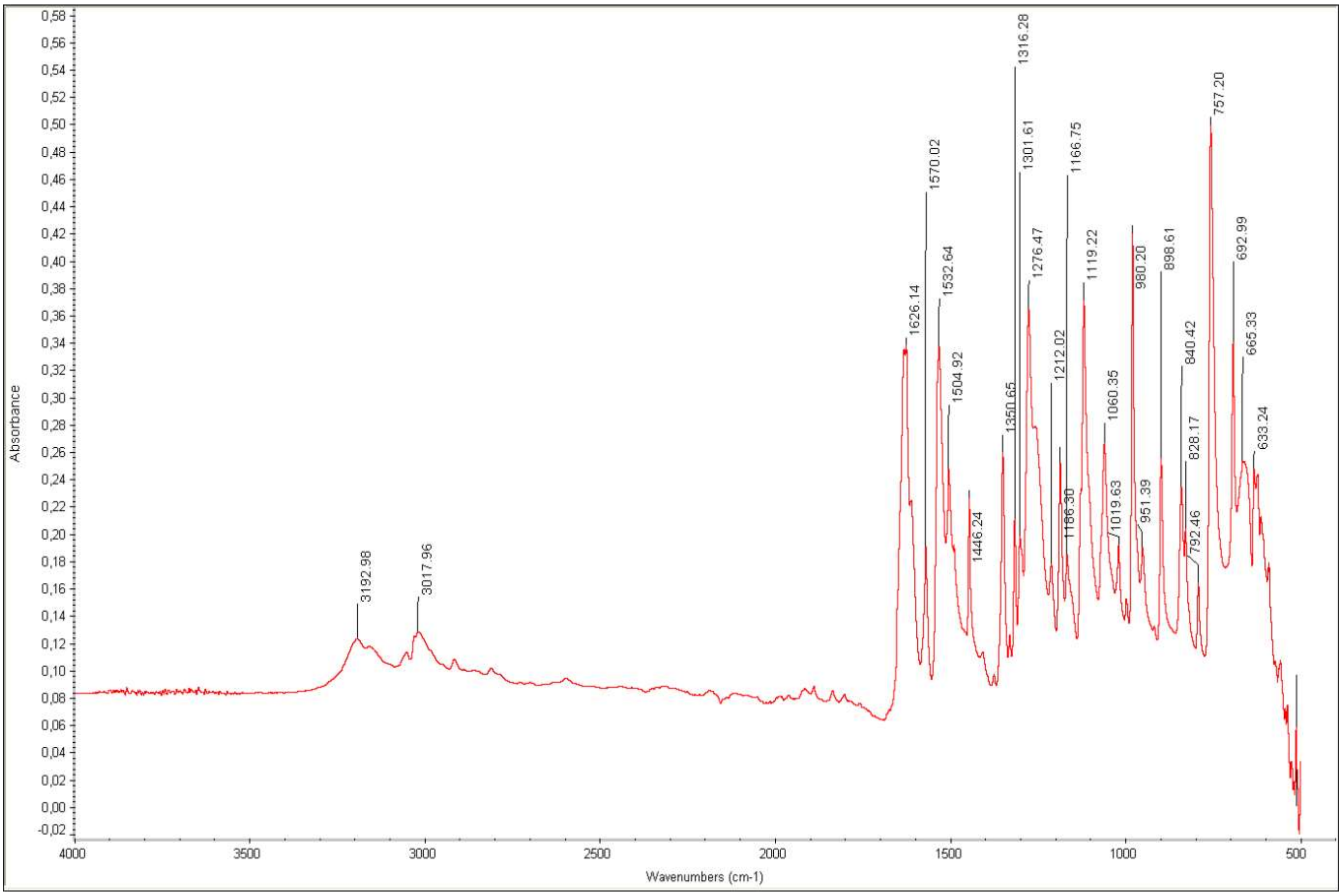


Figure S6. IR spectra of the compound **CA2**.

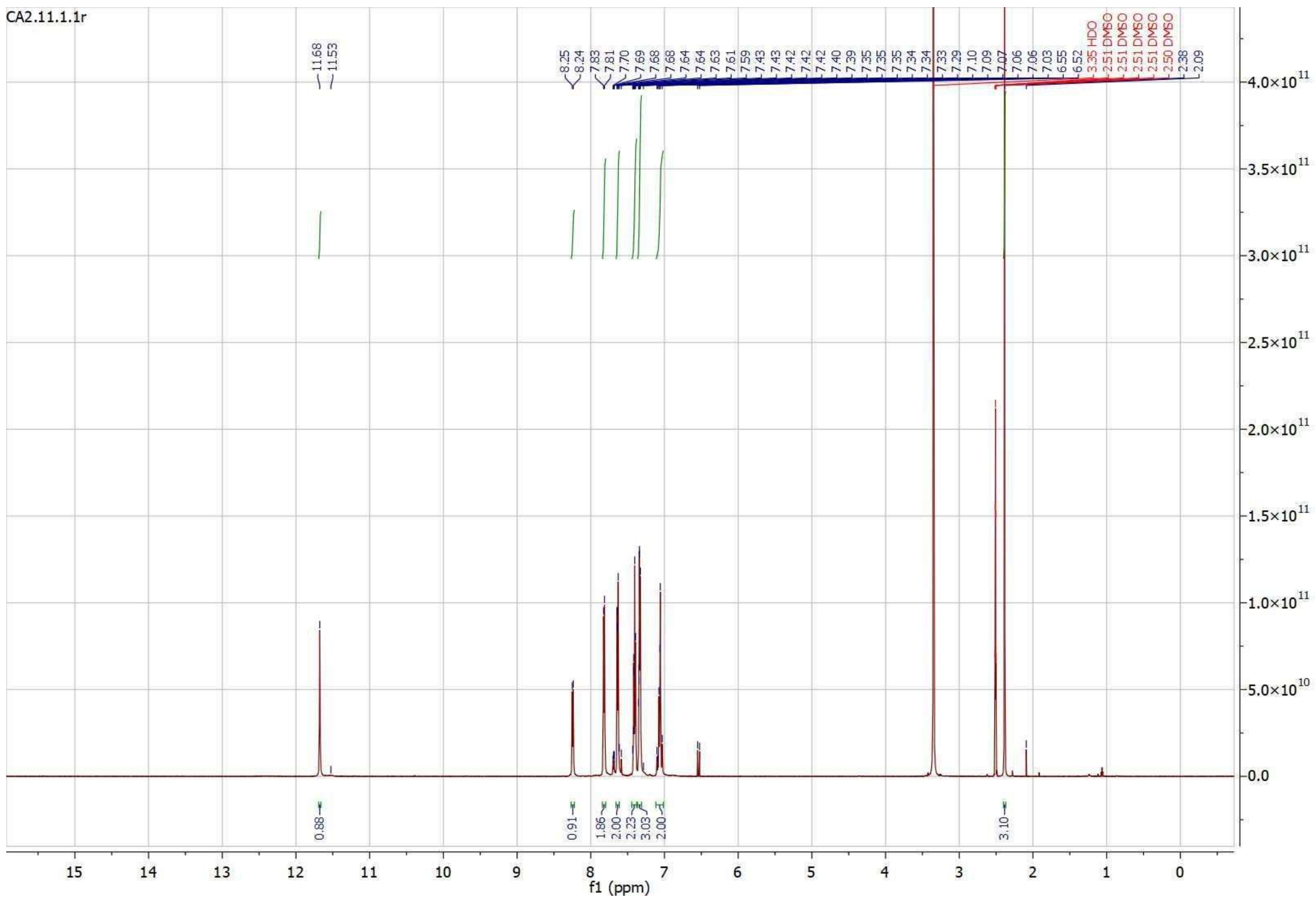


Figure S7. $^{1}H$ NMR spectra of the compound **CA2**.

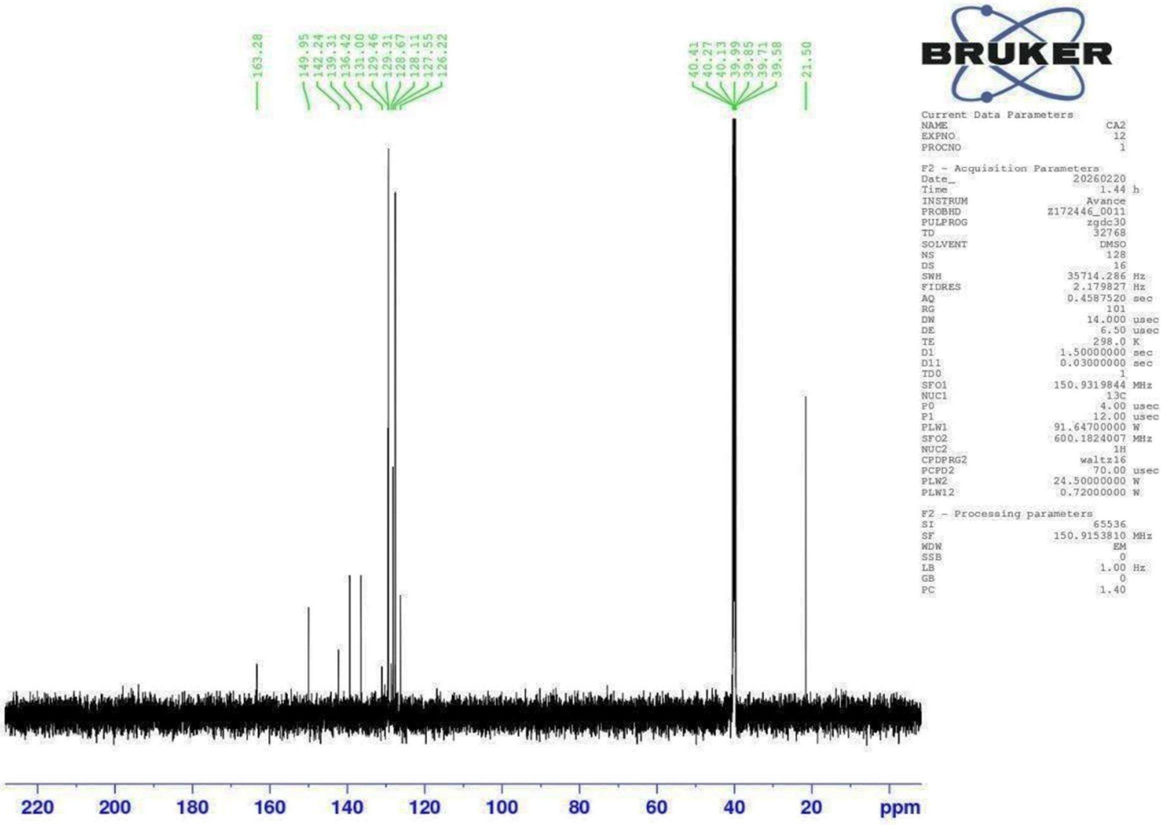


Figure S8. $^{13}$C NMR spectra of the compound **CA2**.

**Compound CA3**

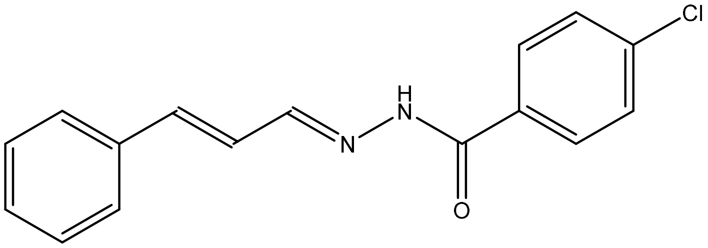


4-chloro-*N'*-((1*E*,2*E*)-3-phenylallylidene)benzohydrazide

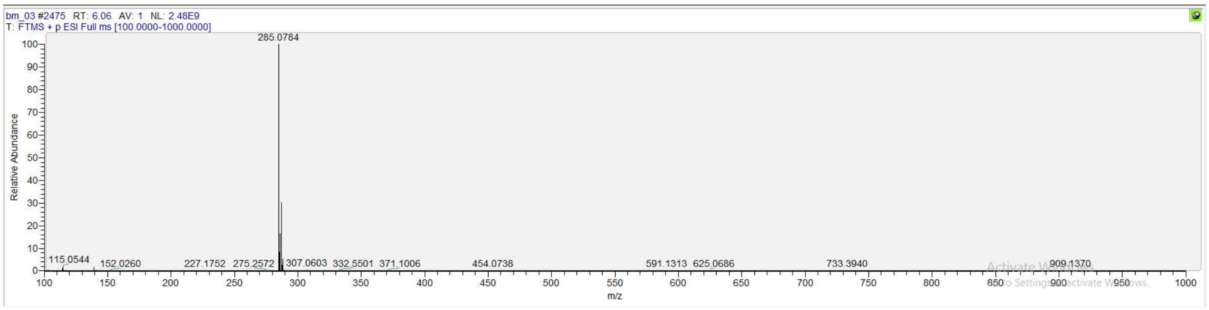


Figure S9. HR ESI–MS spectra of the compound **CA3**.

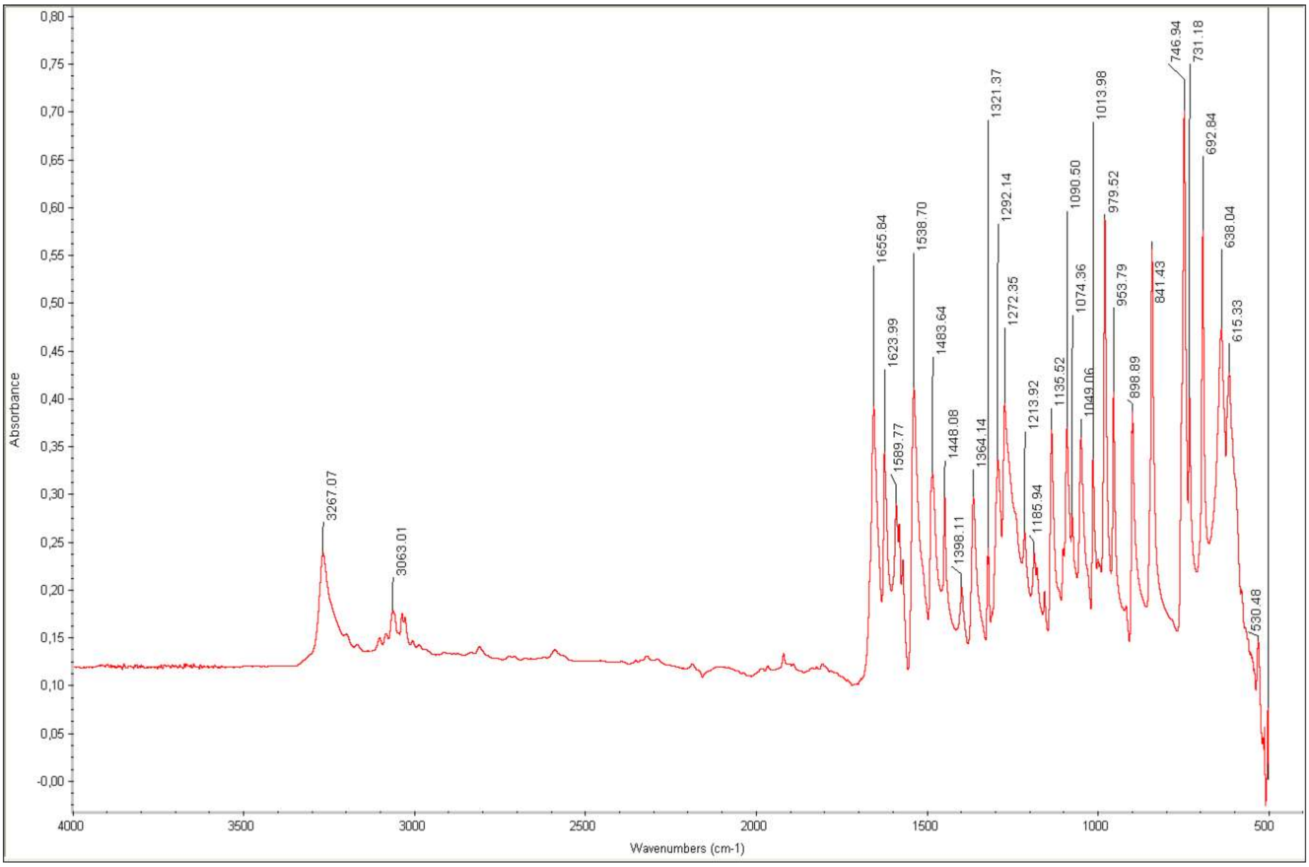


Figure S10. IR spectra of the compound **CA3**.

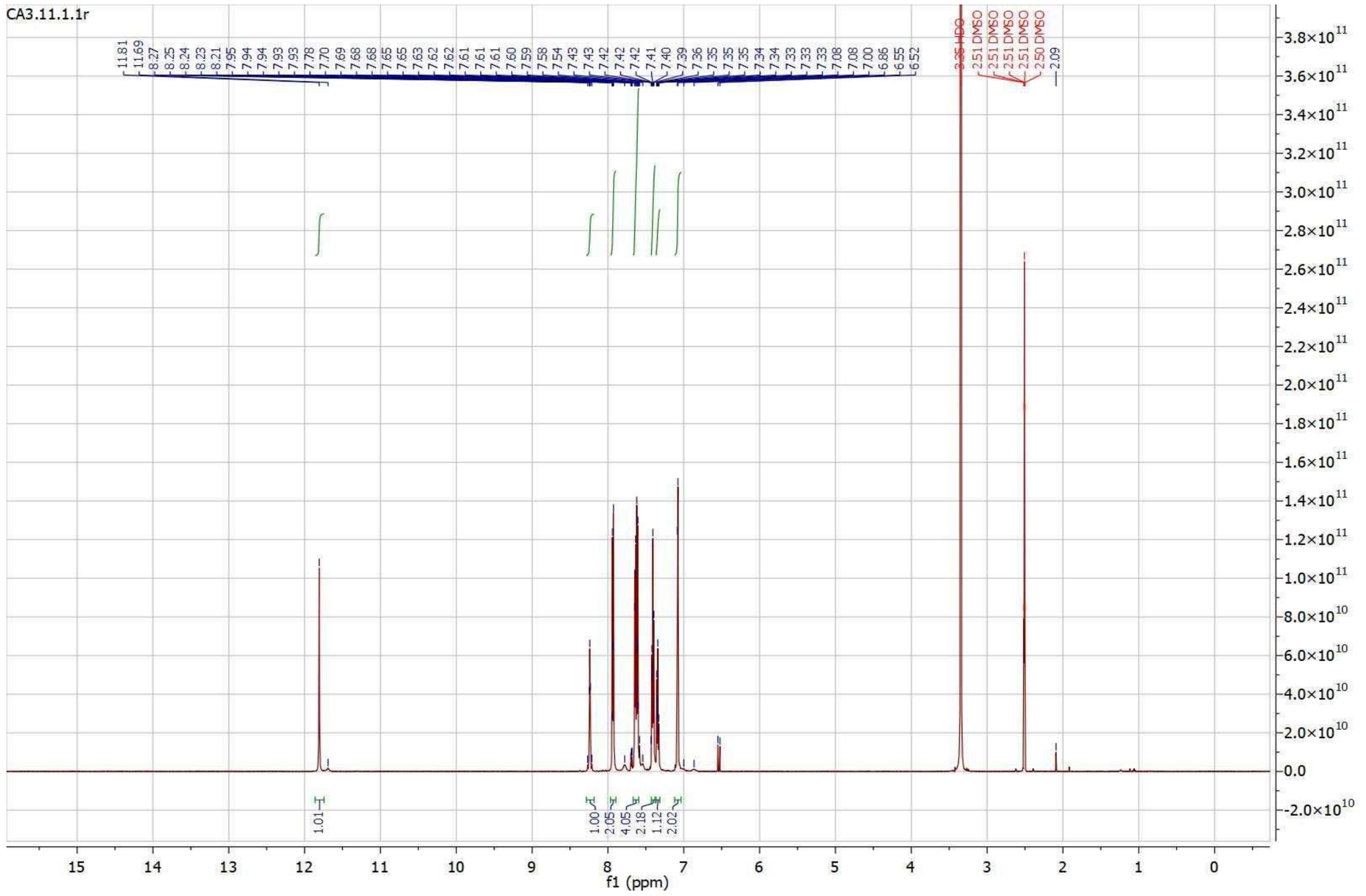


Figure S11. $^{1}$H NMR spectra of the compound **CA3**.

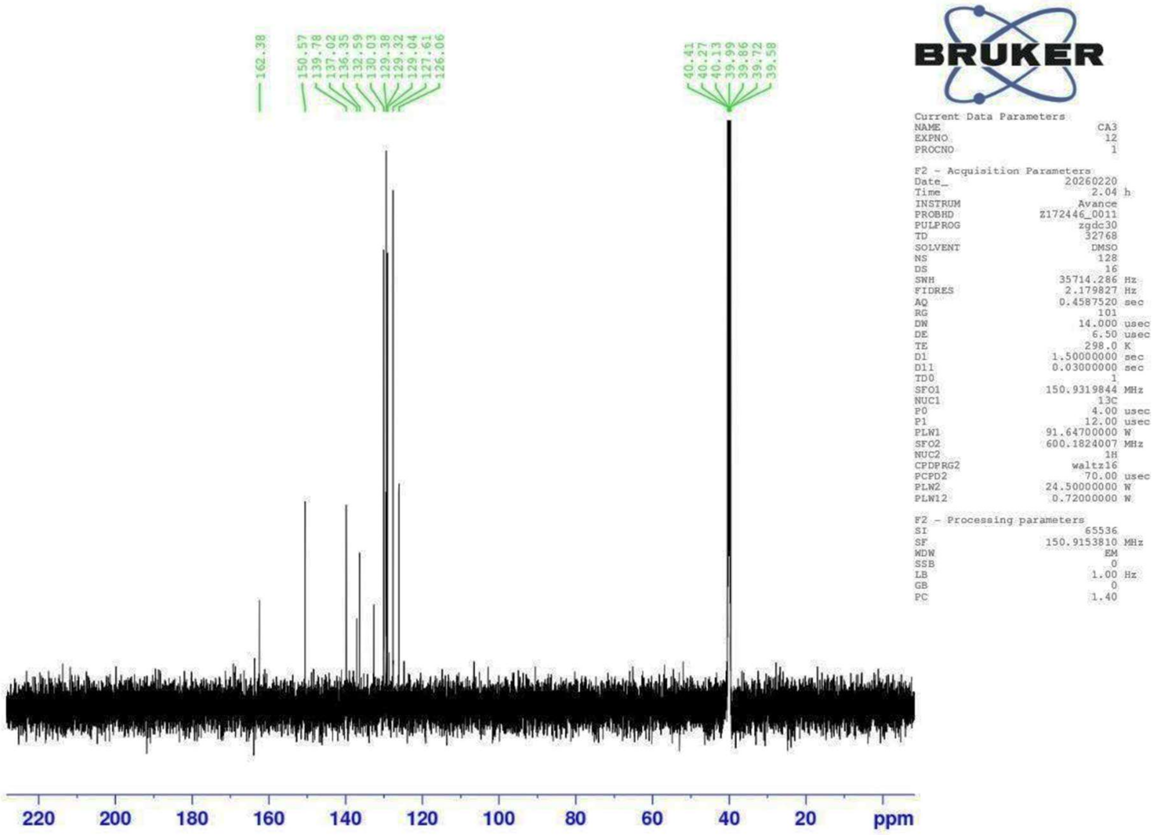


Figure S12. $^{13}C$ NMR spectra of the compound **CA3**.

**Compound CA4**

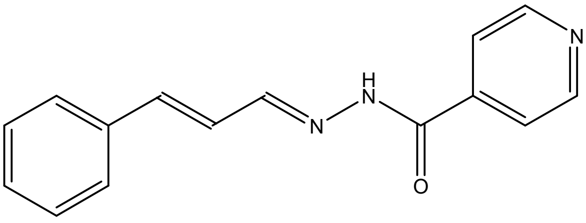


*N'*-((1*E*,2*E*)-3-phenylallylidene)isonicotinohydrazide

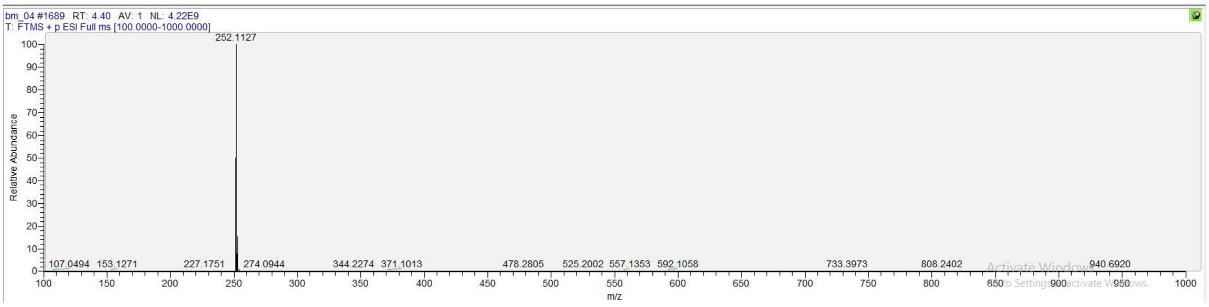


Figure S13. HR ESI–MS spectra of the compound **CA4**.

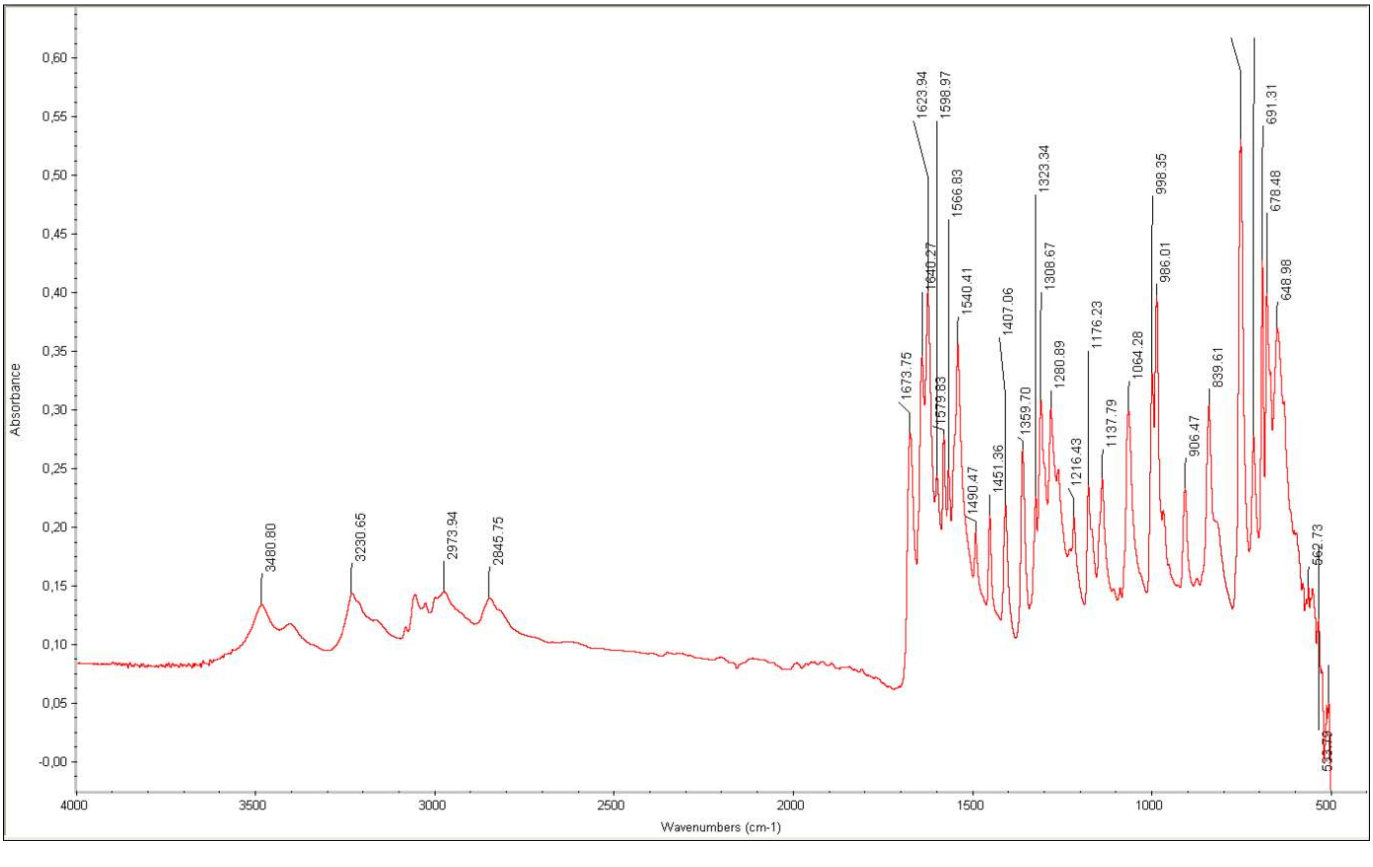


Figure S14. IR spectra of the compound **CA4**.

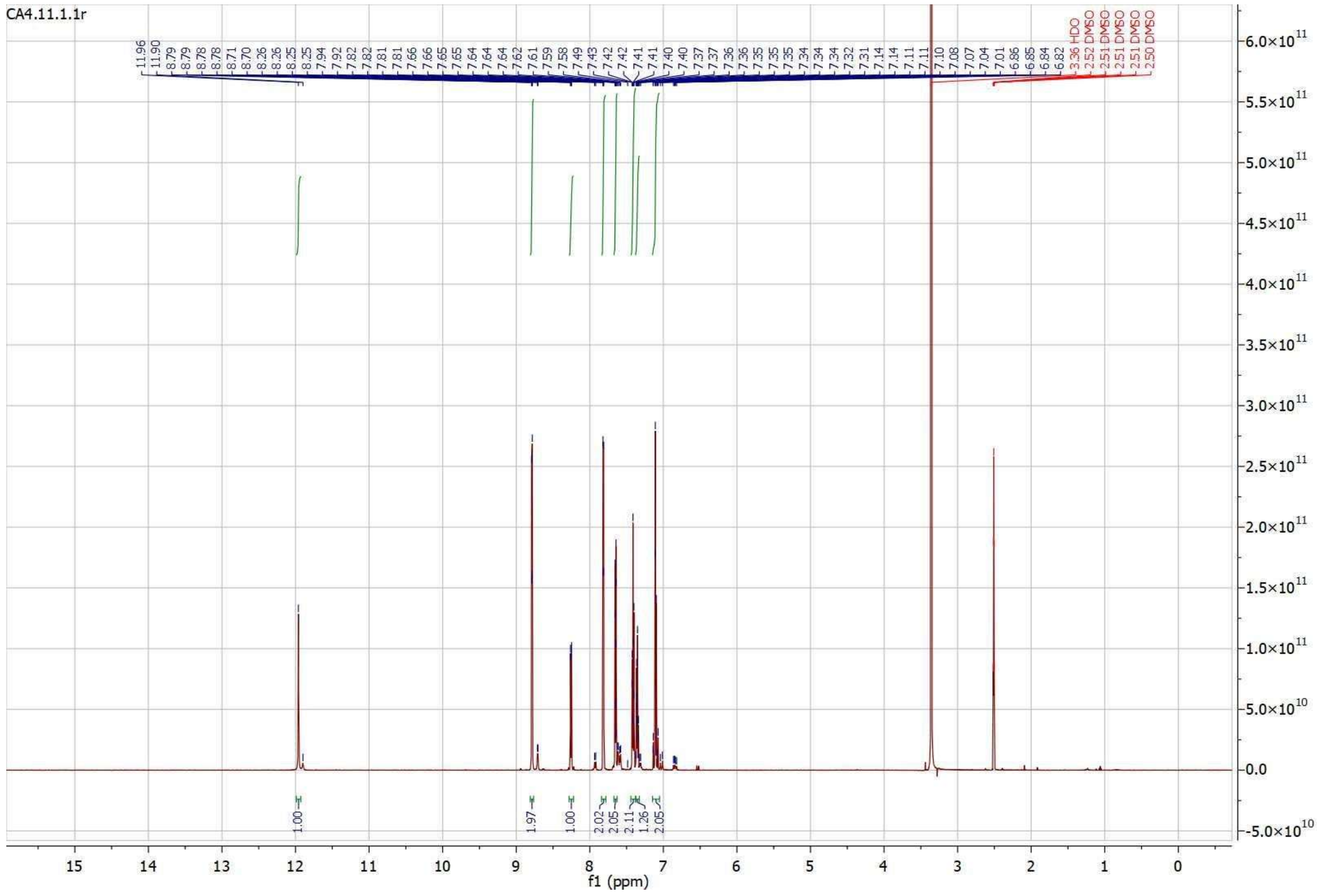


Figure S15. $^{1}$H NMR spectra of the compound **CA4**.

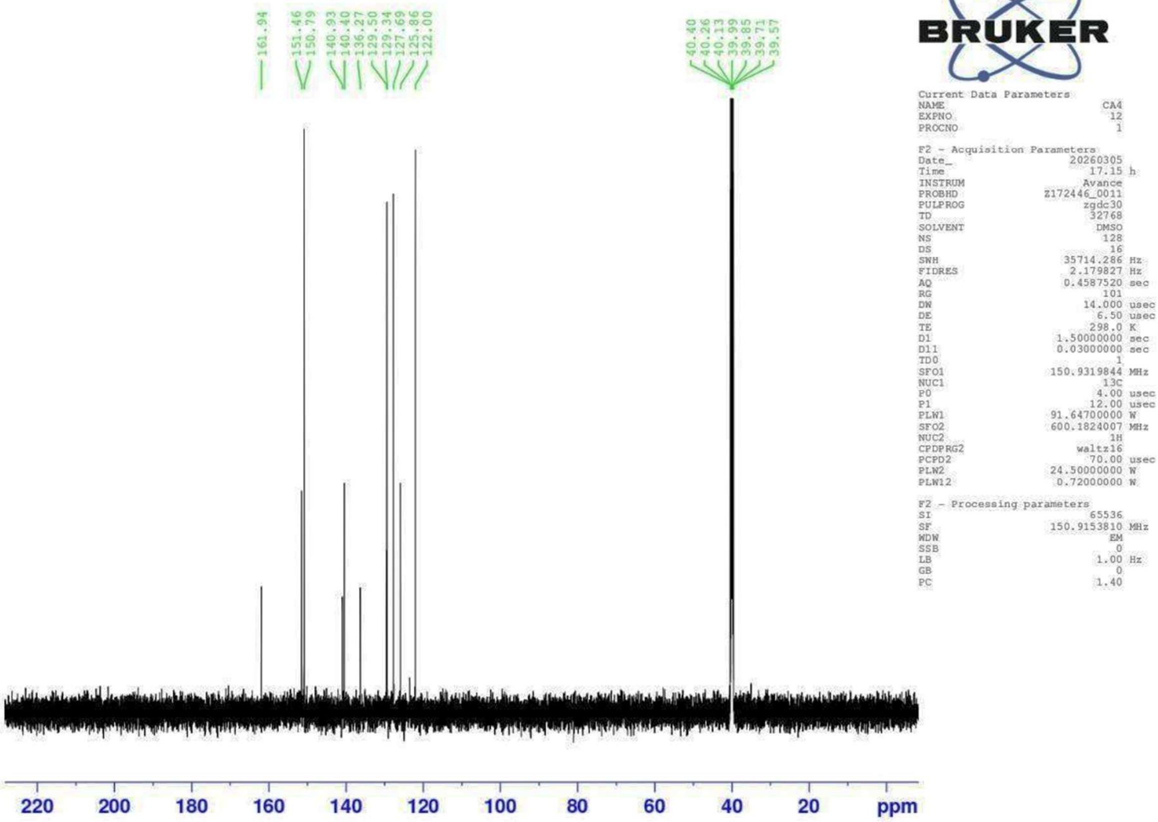


Figure S16. $^{13}$C NMR spectra of the compound **CA4**.

**Compound CA5**

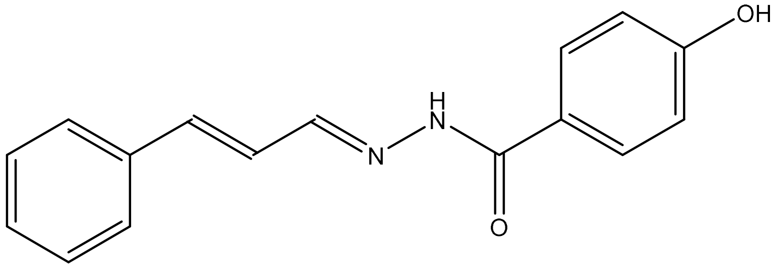


4-hydroxy-*N*'-((1*E*,2*E*)-3-phenylallylidene)benzohydrazide

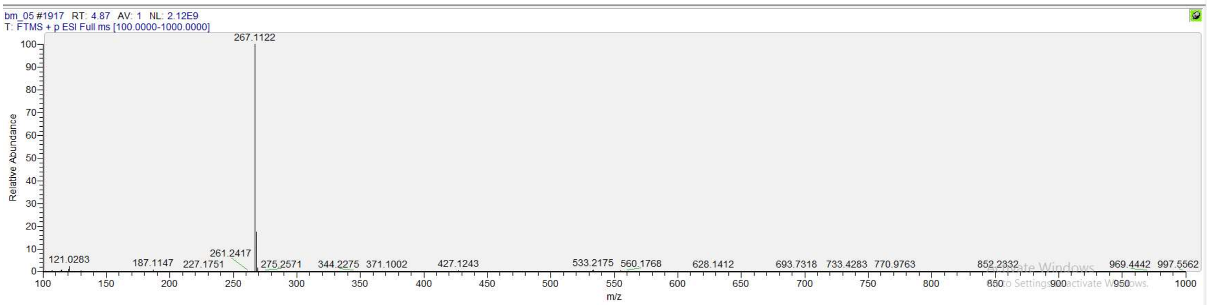


Figure S17. HR ESI–MS spectra of the compound **CA5**.

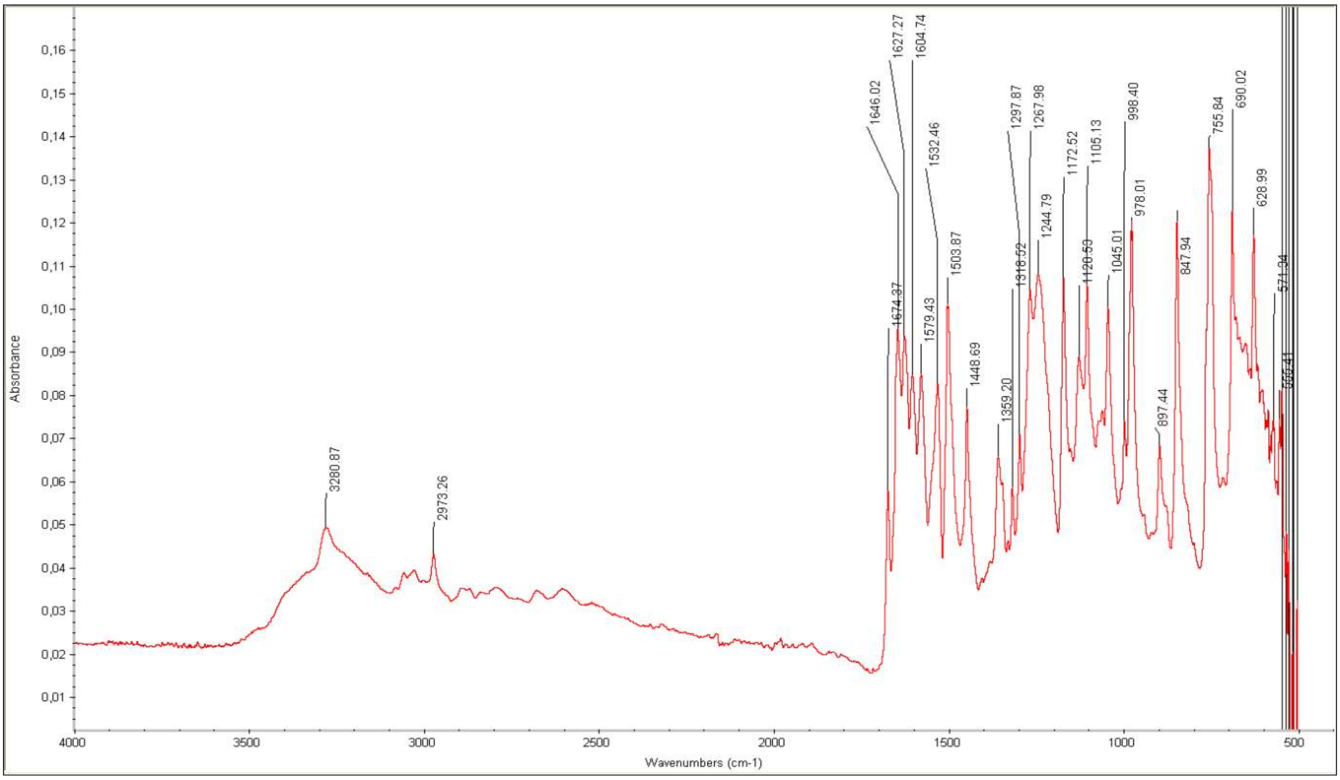


Figure S18. IR spectra of the compound **CA5**.

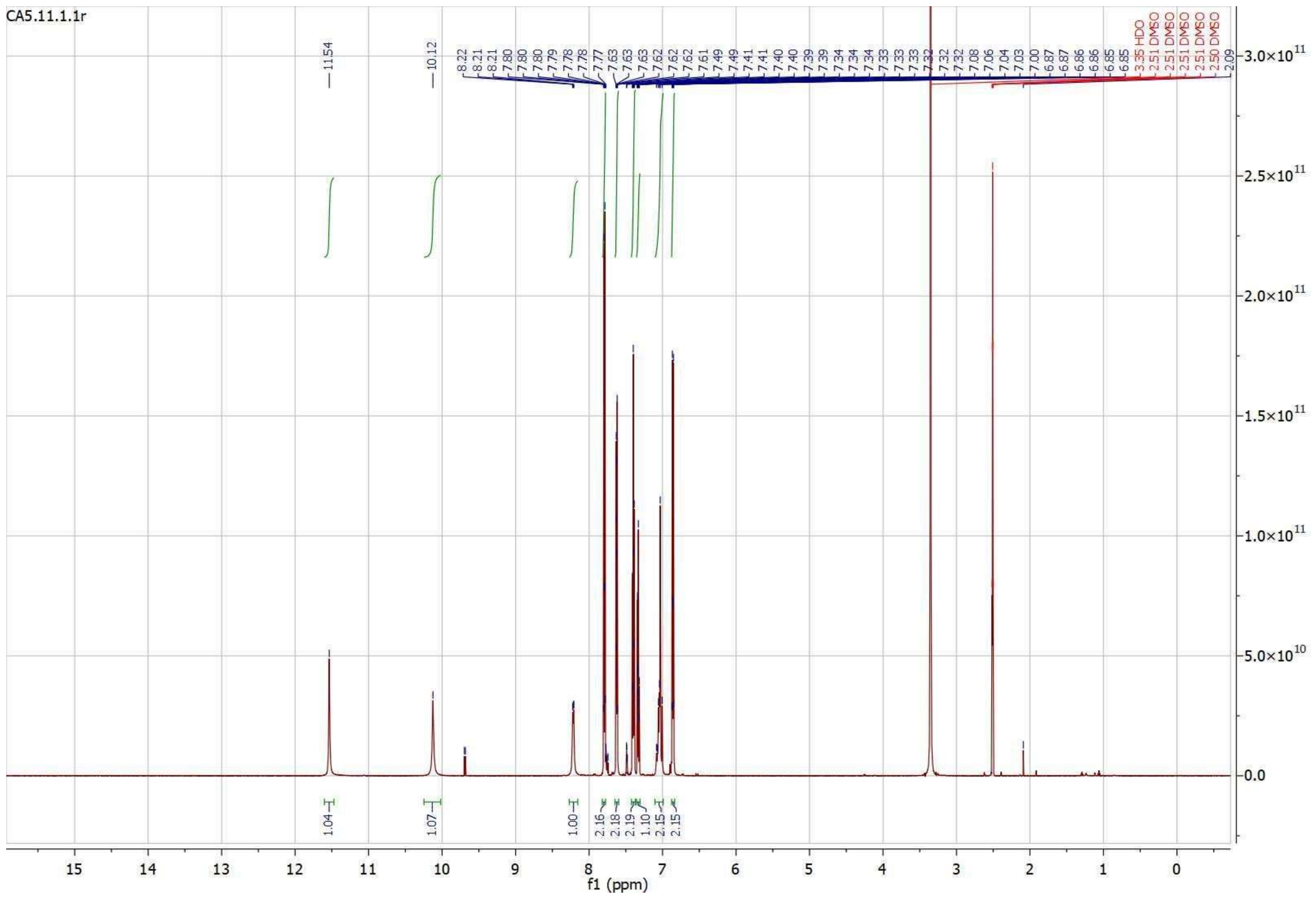


Figure S19. $^{1}$H NMR spectra of the compound **CA5**.

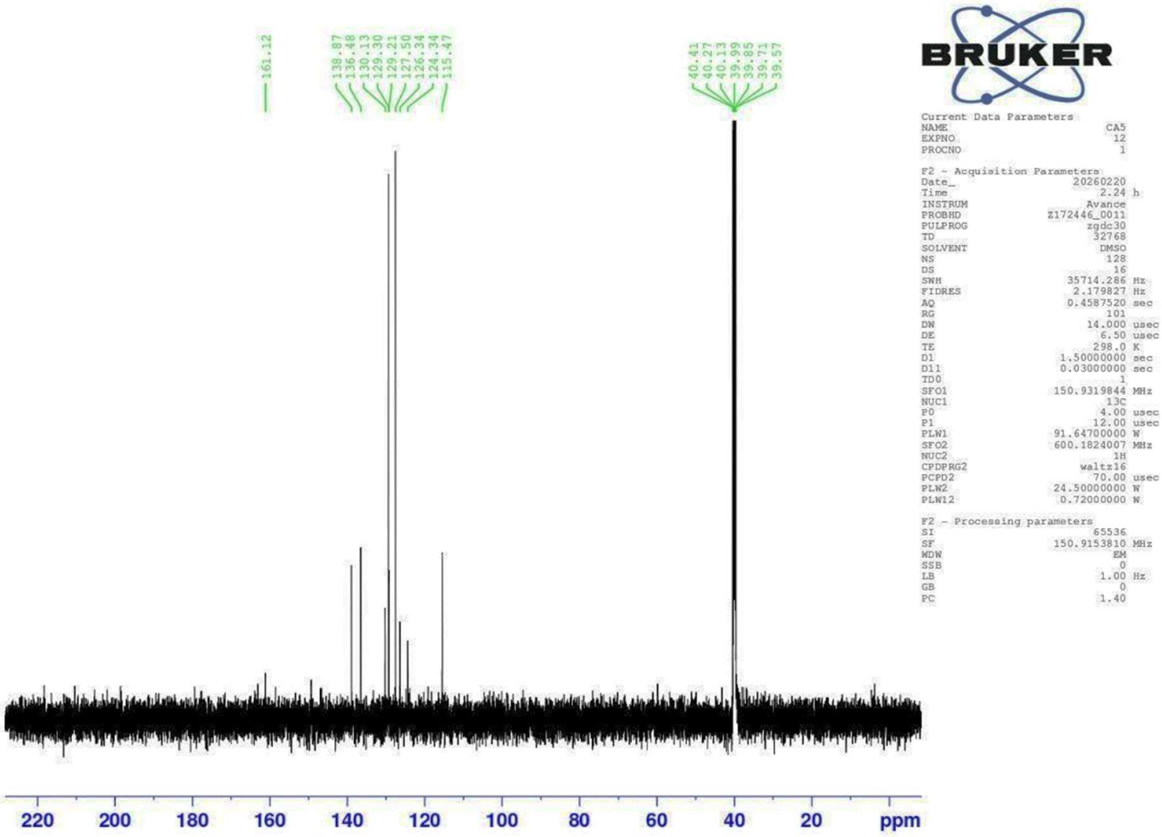

Figure S20. $^{13}$C NMR spectra of the compound **CA5**.

**Compound CA6**

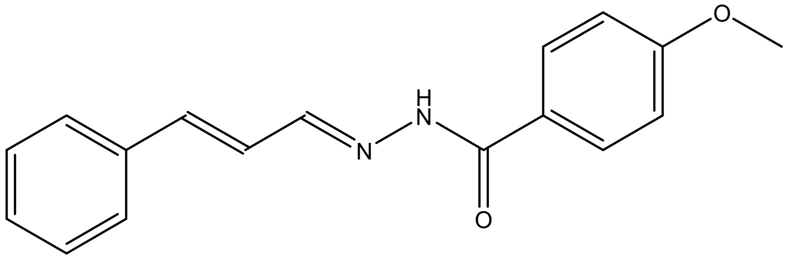


4-methoxy-*N'*-((1*E*,2*E*)-3-phenylallylidene)benzohydrazide

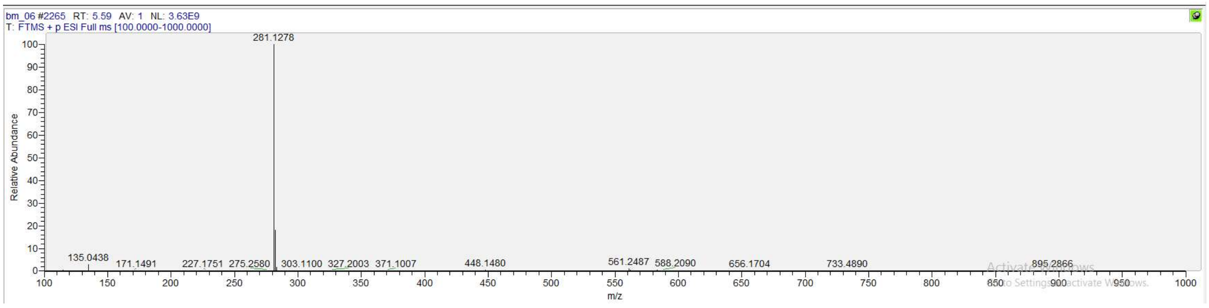


Figure S21. HR ESI–MS spectra of the compound **CA6**.

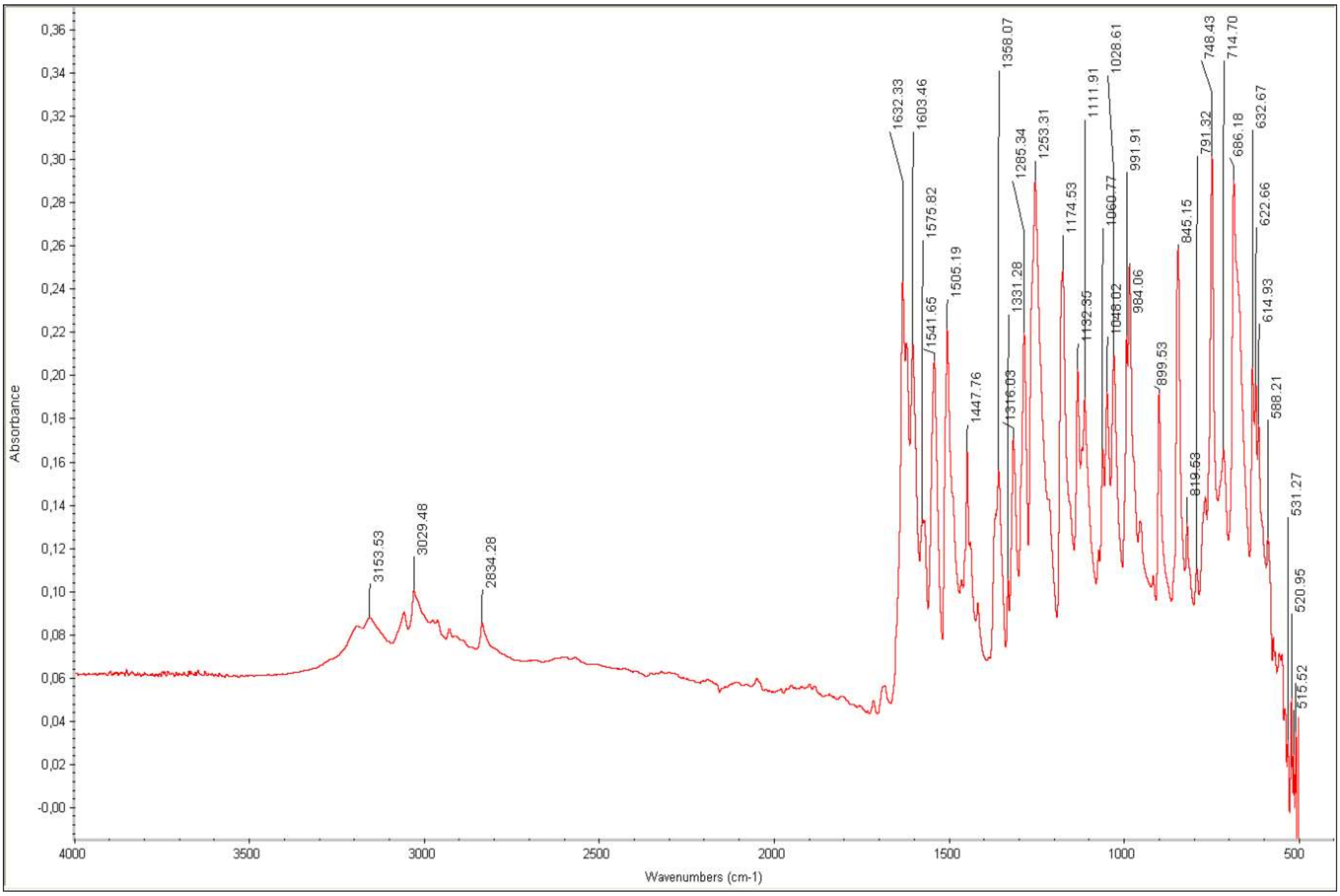


Figure S22. IR spectra of the compound **CA6**.

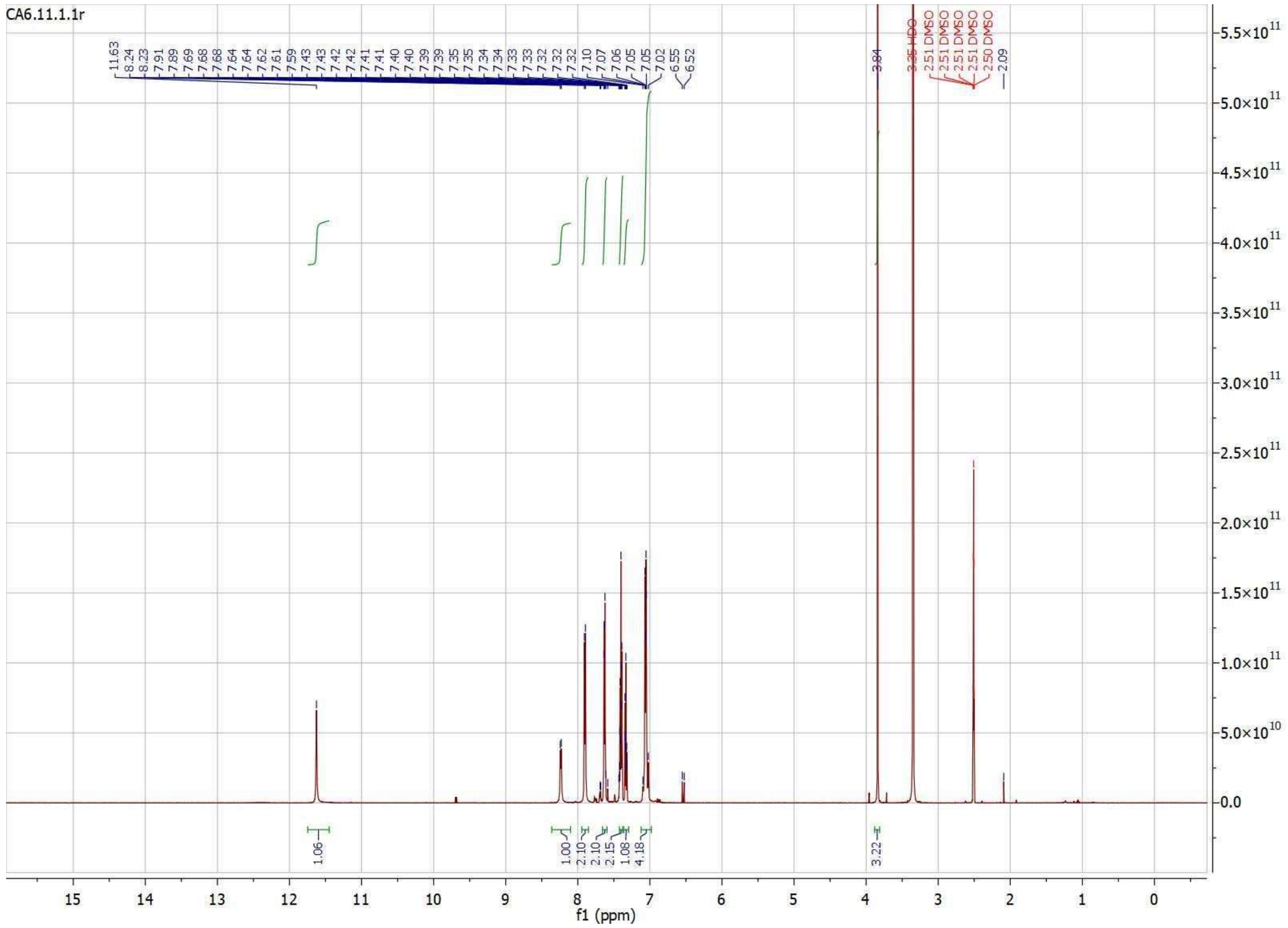


Figure S23. $^{1}$H NMR spectra of the compound **CA6**.

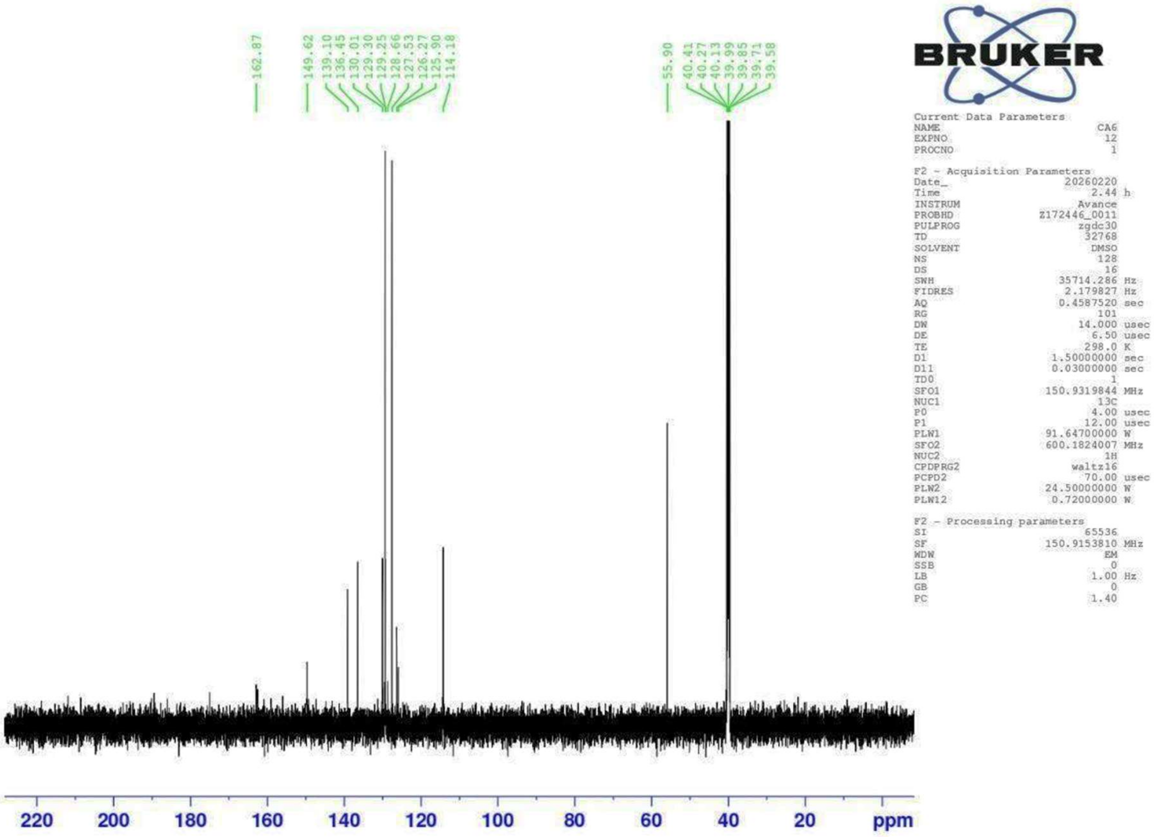

Figure S24. $^{13}$C NMR spectra of the compound **CA6**.

**Compound CA7**

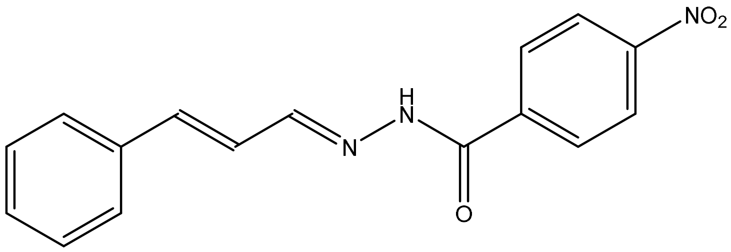


4-nitro-*N'*-((1*E*,2*E*)-3-phenylallylidene)benzohydrazide

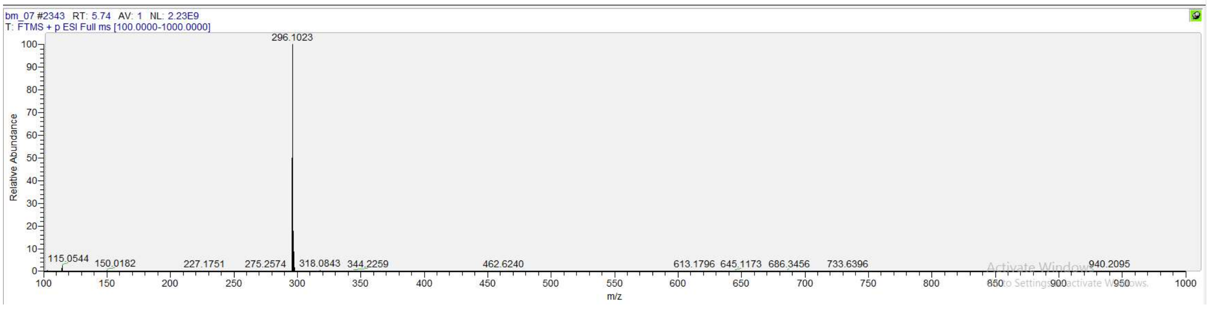


Figure S25. HR ESI–MS spectra of the compound **CA7**.

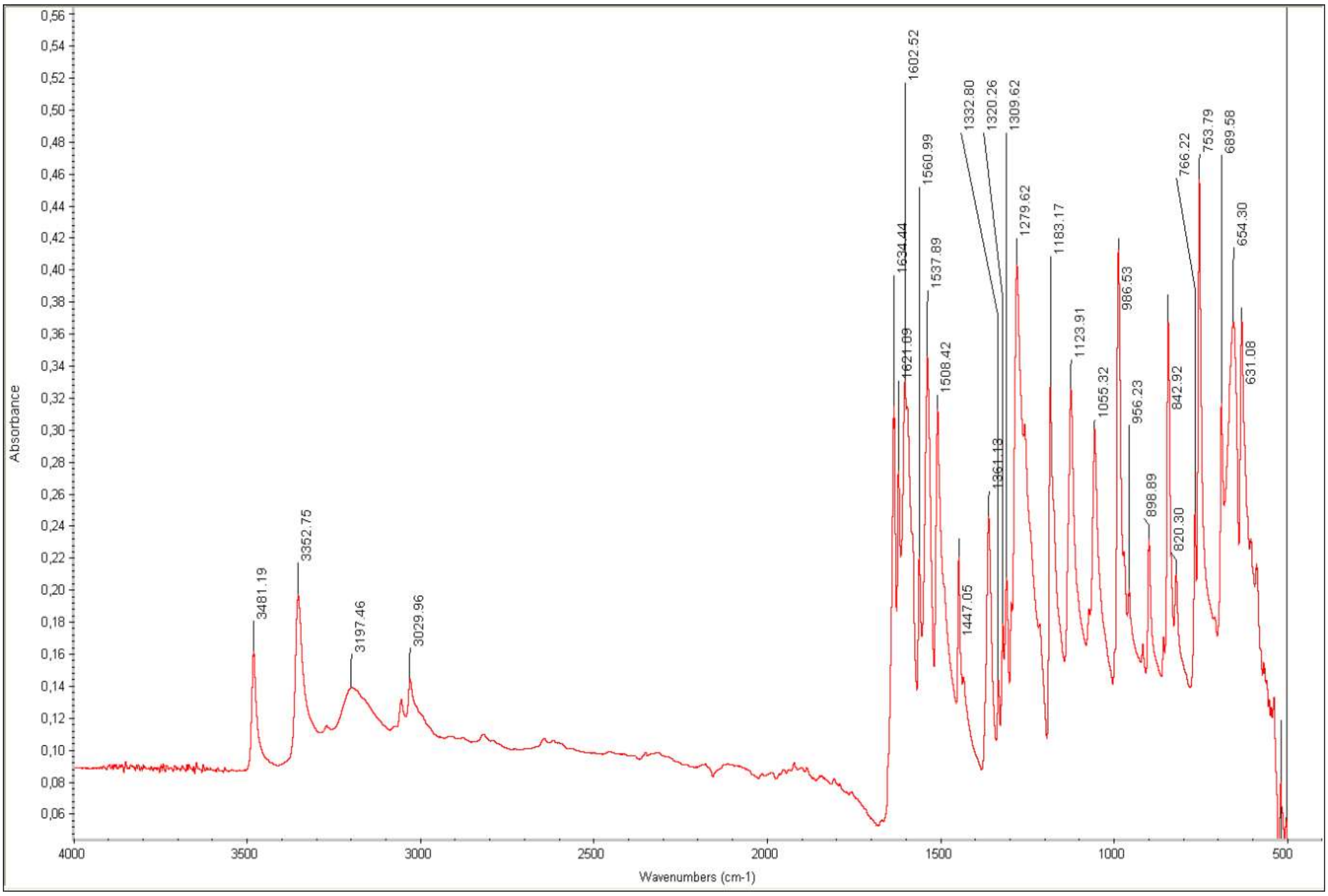


Figure S26. IR spectra of the compound **CA7**.

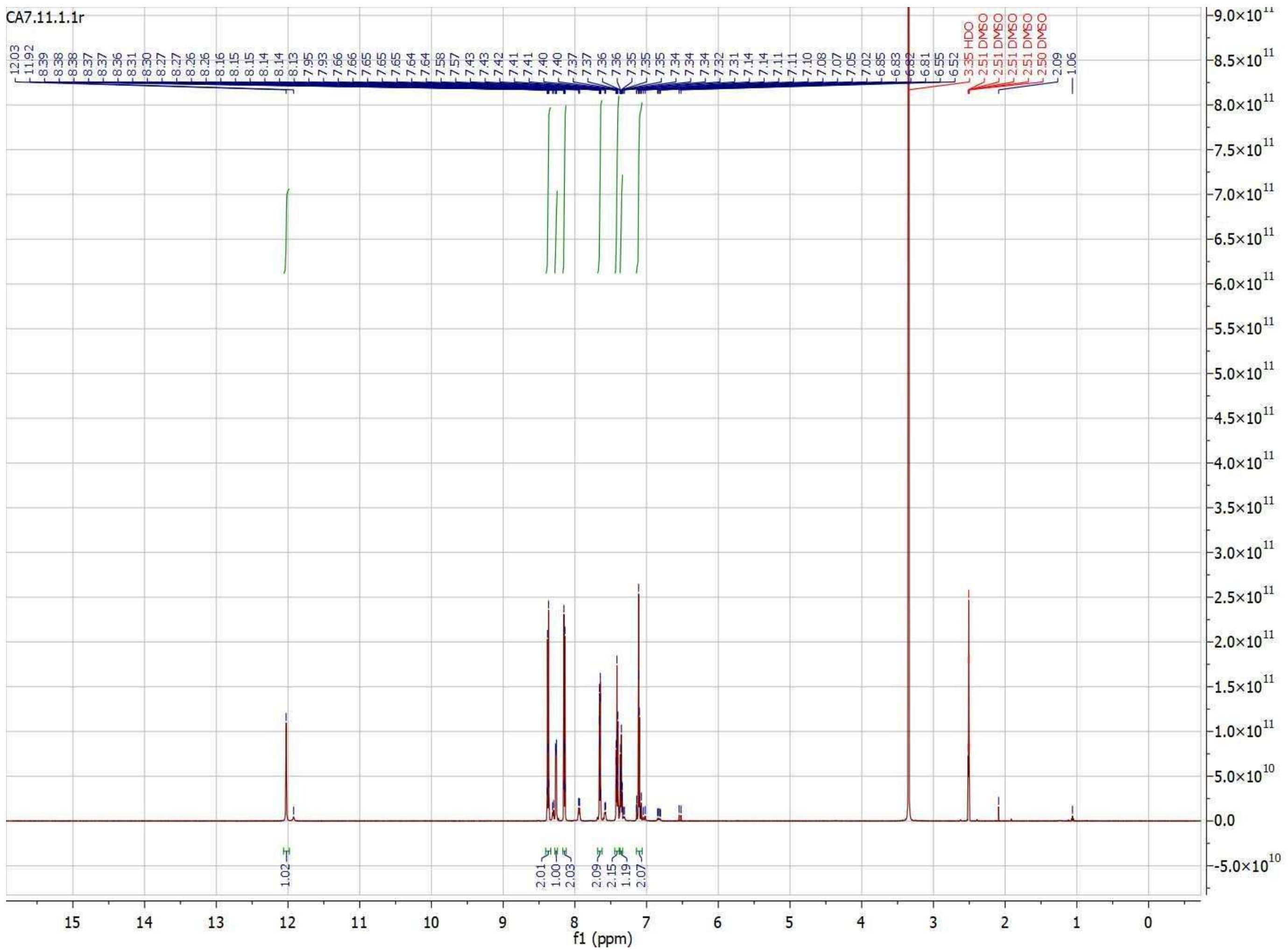

Figure S27. $^{1}$H NMR spectra of the compound **CA7**.

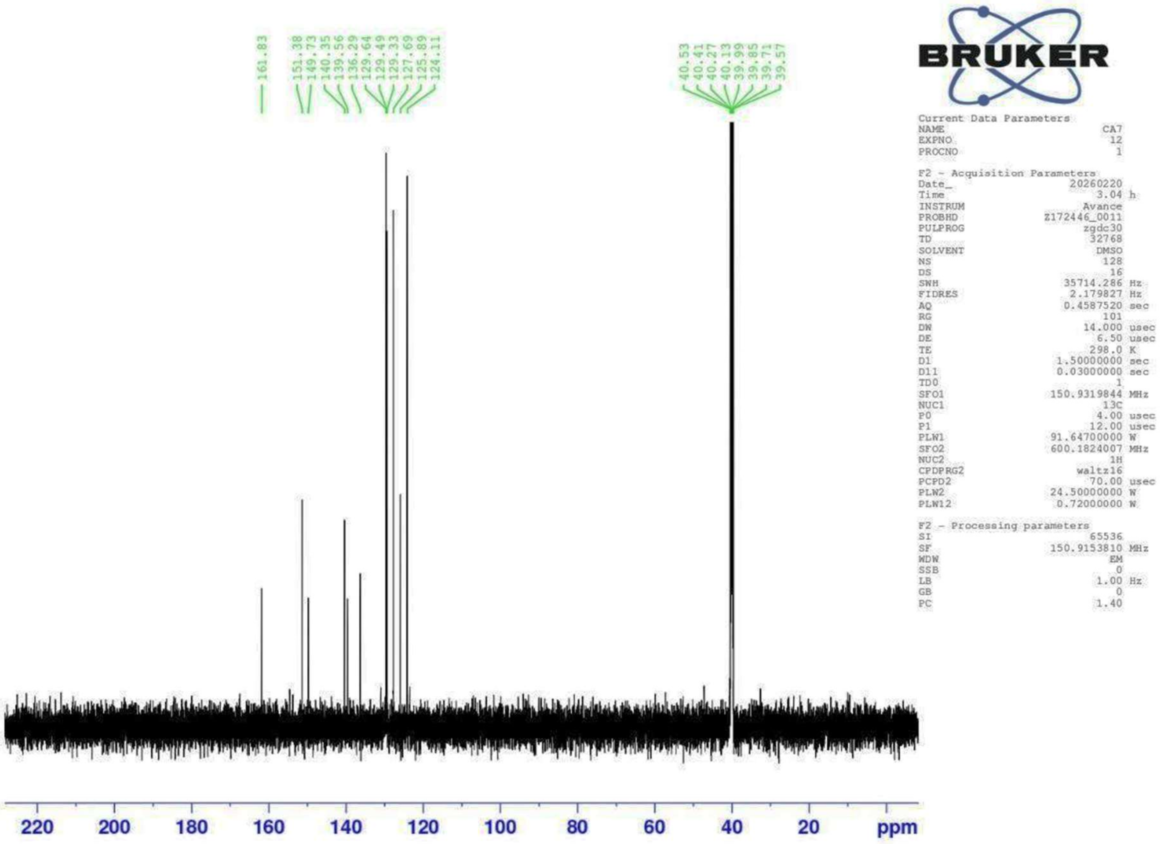


Figure S28. $^{13}C$ NMR spectra of the compound **CA7**.

**Compound CA8**

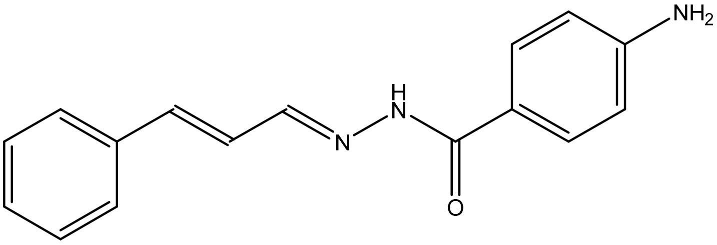


4-amino-*N'*-((1*E*,2*E*)-3-phenylallylidene)benzohydrazide

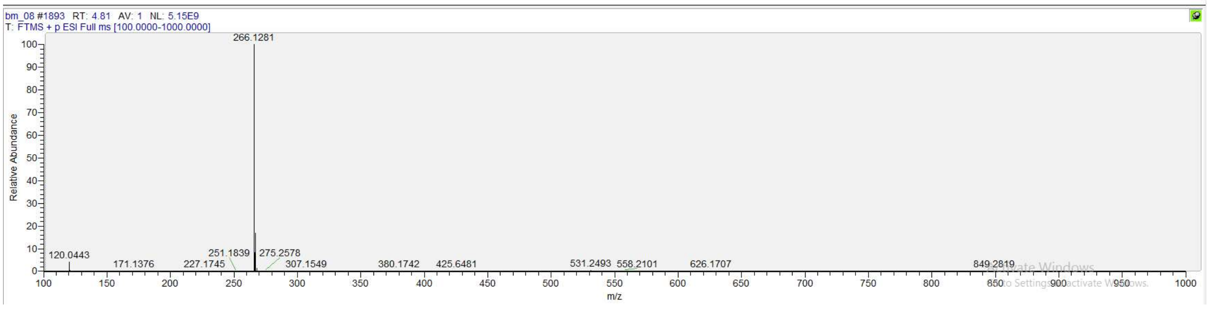


Figure S29. HR ESI–MS spectra of the compound **CA8**.

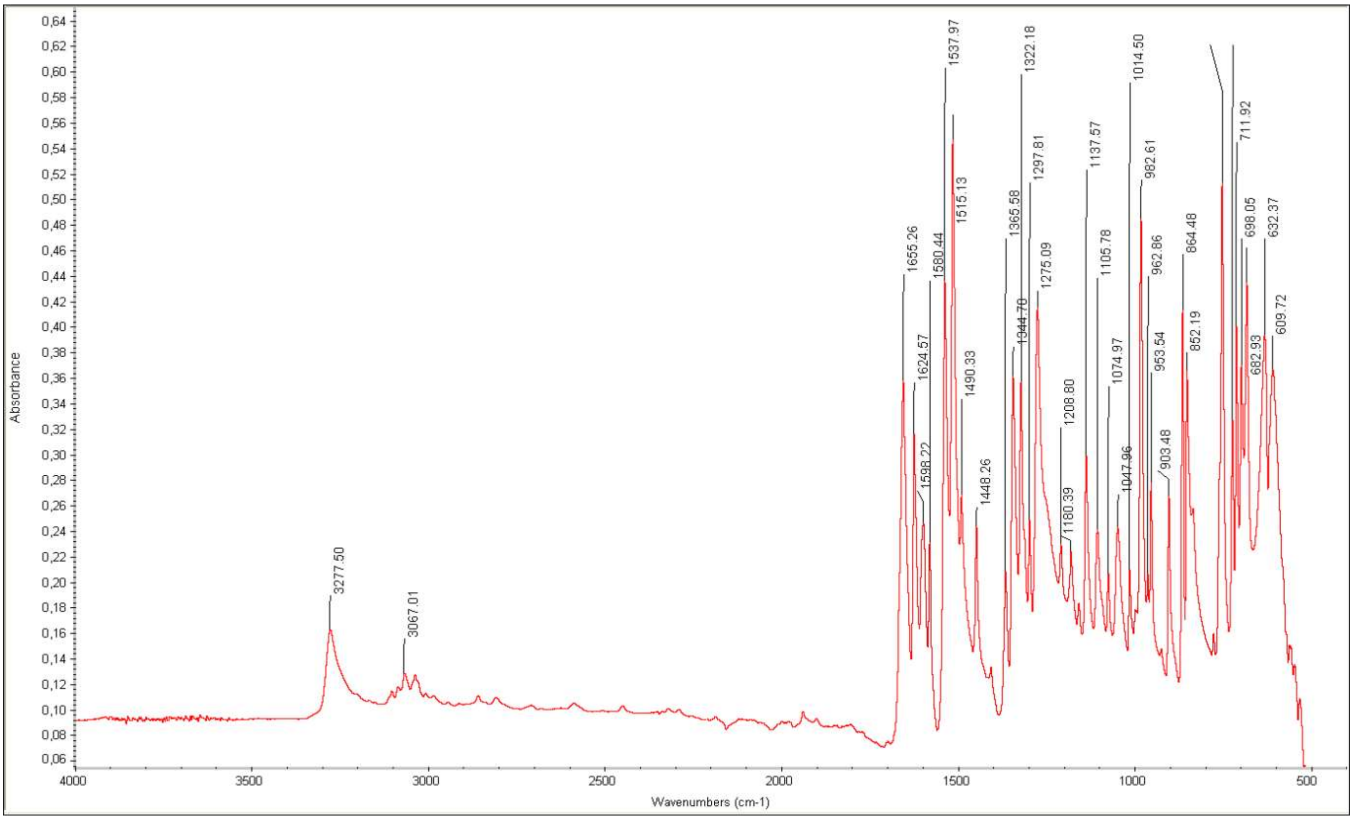


Figure S30. IR spectra of the compound **CA8**.

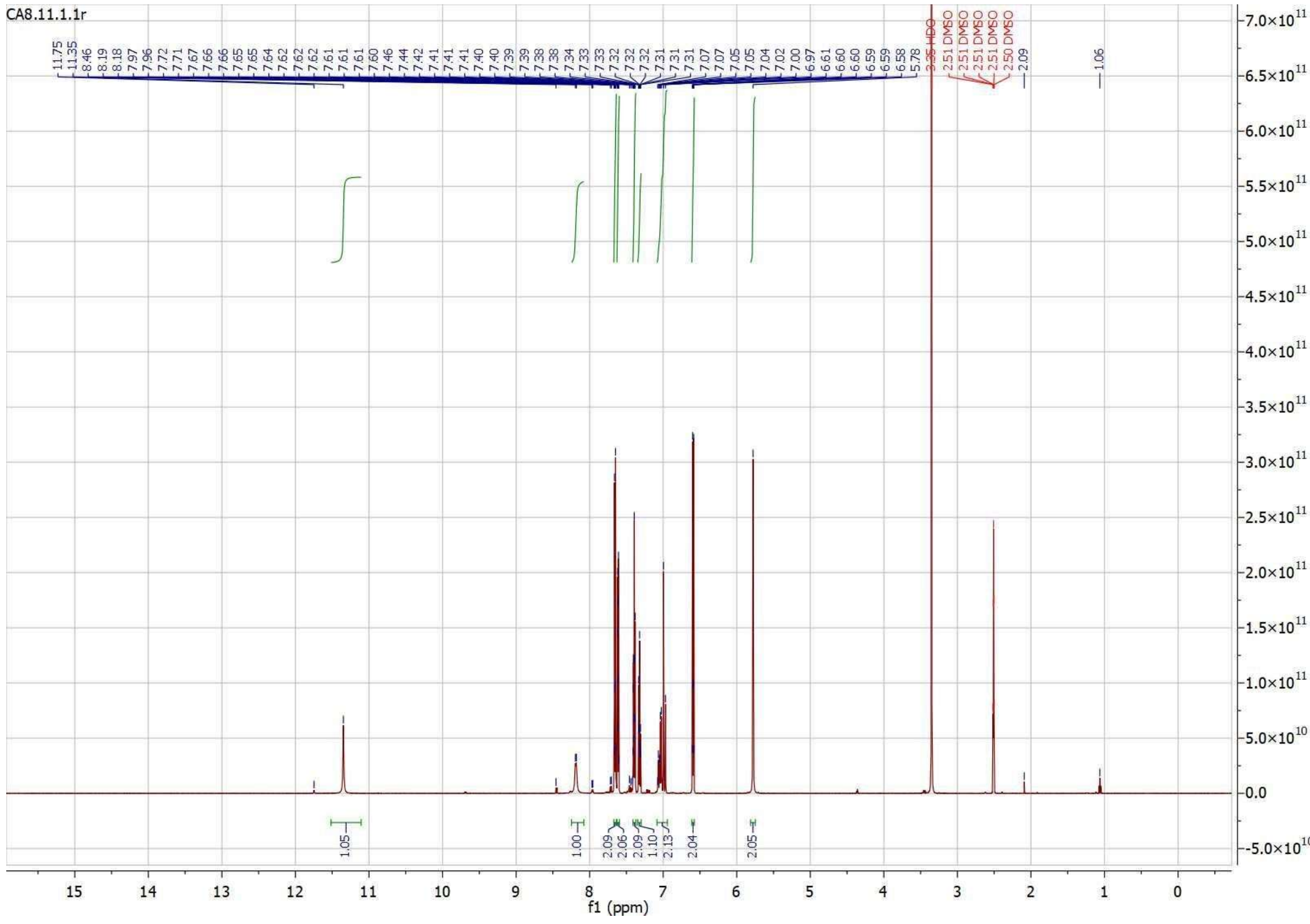


Figure S31. $^{1}$H NMR spectra of the compound **CA8**.

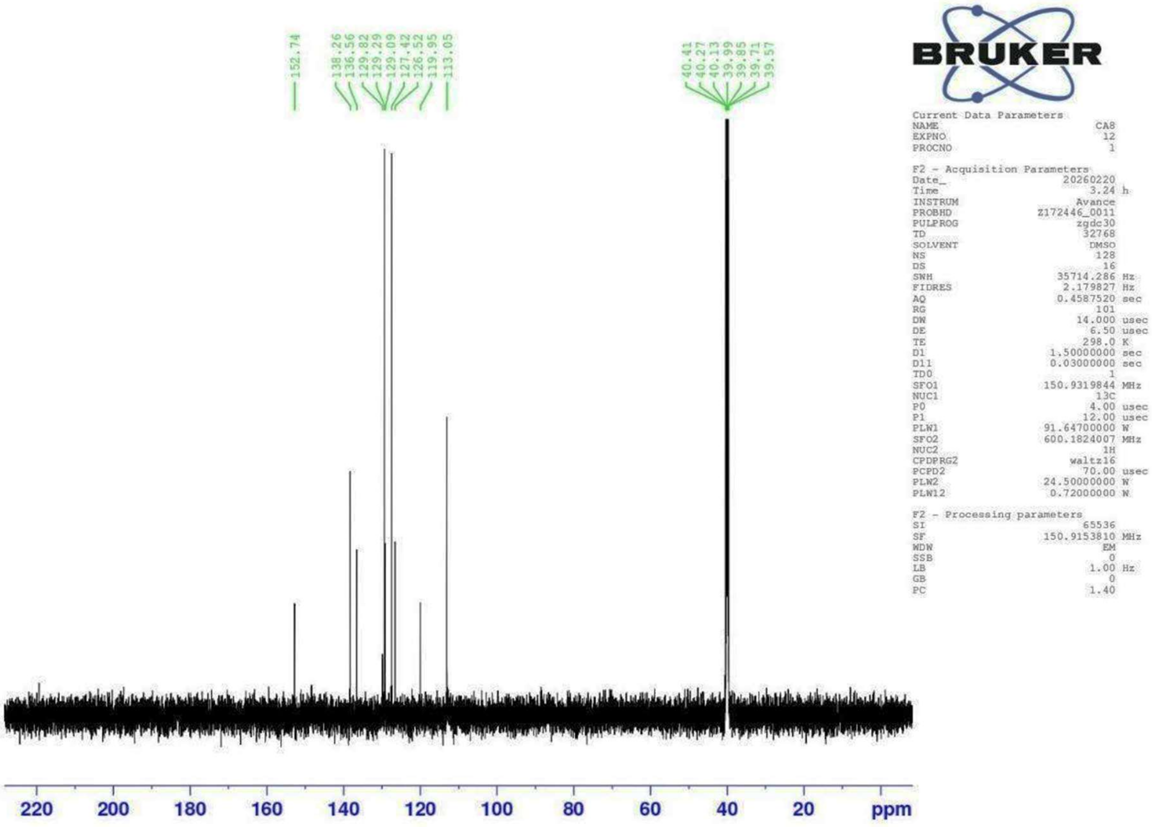


Figure S32. $^{13}$C NMR spectra of the compound **CA8**.
